# Reduced and highly diverse peripheral HIV-1 reservoir in virally suppressed patients infected with non-B HIV-1 strains in Uganda

**DOI:** 10.1186/s12977-022-00587-3

**Published:** 2022-01-15

**Authors:** Samira Joussef-Piña, Immaculate Nankya, Sophie Nalukwago, Joy Baseke, Sandra Rwambuya, Dane Winner, Fred Kyeyune, Keith Chervenak, Bonnie Thiel, Robert Asaad, Curtis Dobrowolski, Benjamin Luttge, Blair Lawley, Cissy M. Kityo, W. Henry Boom, Jonathan Karn, Miguel E. Quiñones-Mateu

**Affiliations:** 1 Departments of Molecular Biology and Microbiology, Case Western Reserve University, Cleveland, OH USA; 2 Center for AIDS Research Uganda Laboratories, Joint Clinical Research Centre, Kampala, Uganda; 3 Departments of Medicine, Case Western Reserve University, Cleveland, OH USA; 4 Department of Microbiology and Immunology, School of Biomedical Sciences, University of Otago, 720 Cumberland Street, P.O. Box 56, Dunedin, New Zealand; 5 Webster Centre for Infectious Diseases, University of Otago, Dunedin, New Zealand

**Keywords:** HIV, Reservoir, Uganda, Subtype, Co-infection

## Abstract

**Background:**

Our understanding of the peripheral human immunodeficiency virus type 1 (HIV-1) reservoir is strongly biased towards subtype B HIV-1 strains, with only limited information available from patients infected with non-B HIV-1 subtypes, which are the predominant viruses seen in low- and middle-income countries (LMIC) in Africa and Asia.

**Results:**

In this study, blood samples were obtained from well-suppressed ART-experienced HIV-1 patients monitored in Uganda (n = 62) or the U.S. (n = 50), with plasma HIV-1 loads < 50 copies/ml and CD4^+^ T-cell counts > 300 cells/ml. The peripheral HIV-1 reservoir, i.e., cell-associated HIV-1 RNA and proviral DNA, was characterized using our novel deep sequencing-based EDITS assay. Ugandan patients were slightly younger (median age 43 vs 49 years) and had slightly lower CD4^+^ counts (508 vs 772 cells/ml) than U.S. individuals. All Ugandan patients were infected with non-B HIV-1 subtypes (31% A1, 64% D, or 5% C), while all U.S. individuals were infected with subtype B viruses. Unexpectedly, we observed a significantly larger peripheral inducible HIV-1 reservoir in U.S. patients compared to Ugandan individuals (48 vs. 11 cell equivalents/million cells, *p* < 0.0001). This divergence in reservoir size was verified measuring proviral DNA (206 vs. 88 cell equivalents/million cells, *p* < 0.0001). However, the peripheral HIV-1 reservoir was more diverse in Ugandan than in U.S. individuals (8.6 vs. 4.7 p-distance, *p* < 0.0001).

**Conclusions:**

The smaller, but more diverse, peripheral HIV-1 reservoir in Ugandan patients might be associated with viral (e.g., non-B subtype with higher cytopathicity) and/or host (e.g., higher incidence of co-infections or co-morbidities leading to less clonal expansion) factors. This highlights the need to understand reservoir dynamics in diverse populations as part of ongoing efforts to find a functional cure for HIV-1 infection in LMICs.

**Supplementary Information:**

The online version contains supplementary material available at 10.1186/s12977-022-00587-3.

## Background

Although widespread global access to antiretroviral therapy (ART) has led to considerable reductions in HIV/AIDS morbidity and mortality [1, 2], the HIV-1 epidemic continues to affect millions of people worldwide, with the highest burden in low- and middle-income countries (LMIC) [3, 4], Unfortunately, while ART effectively controls HIV-1 replication in plasma to below current detection levels, HIV-1 persists mostly in latently-infected effector and transitional memory CD4^+^ T cells, with minimal decay despite prolonged ART [5–7]. This pool of latent proviruses is quickly established after infection, usually leading to a steady source of potentially replication-competent HIV-1 that persists indefinitely even in well suppressed cART-treated people living with HIV-1 (PLWH) [8]. Thus, HIV-1 post-integration latency represents the main obstacle to completely eradicate the virus from infected individuals [8, 9].

In the absence of HIV-1 transcription and virus production, the host immune system is not able to recognize latently HIV-infected cells or target them for elimination [8, 10]. To circumvent this problem, one avenue to eradicate the latent HIV-1 reservoir has been the “shock and kill” strategy, which involves activation of latent HIV-1 by latency reversal agents (LRA) in the presence of ART in conjunction with immunological approaches designed to purge the reactivated cells [11–13]. Numerous pre-clinical studies have used multitude of strategies, mainly based on LRAs, to re-activate the HIV-1 reservoir [3, 8, 13, 14]; however, to date no LRA-based therapy—not even the promising histone deacetylase inhibitors (HDACIs)—have been able to successfully perturb the HIV-1 reservoir in human clinical trials [8, 15]. It is clear that an incomplete understanding of the molecular nature of proviral latency has hampered progress in this area [16]. Numerous complementary approaches are also being explored for a functional HIV-1 cure, such as stand-alone immunological enhancements, including broadly neutralizing antibodies, direct targeting of the genome with CRISPRs, and induction of permanent epigenetic silencing [17].

Any successful effort to eradicate latently HIV-infected cells needs to be supported by accurate, reproducible and informative measurements of the proviral reservoir. Many competing methods have been developed to quantify the HIV-1 reservoir and measure the efficacy of strategies to functionally cure PLWH [15, 18, 19]. Effective assays that measure the latent HIV-1 reservoir need exquisite sensitivity, the capacity to distinguish between potentially replication-competent and defective proviruses, and the ability to accurately measure signals resulting from partial proviral activation. The original method for measurement of the latent HIV-1 reservoir was the quantitative viral outgrowth assay (Q-VOA) [20]. This assay uses uninfected but activated, or transformed cells, to exponentially amplify replication-competent HIV-1 released from latently infected cells after T-cell activation [20–22]. Unfortunately, Q-VOA is costly and labor intensive, only semi-quantitative and appears to miss many replication-competent proviruses that may not be induced following a single round of T cell activation or are lost due to cytopathic effects arising from ex vivo induction [15].

The search for an alternative to Q-VOA has led to the development of a series of molecular and/or cell culture-based assays aimed to simplify the quantitation of the HIV-1 reservoir [15, 18, 19]. PCR-based assays that measure total proviral and integrated DNA are technically easier to perform, quantitatively more rigorous and the methodology is well established [23–26]. Unfortunately, interpretation of proviral DNA measurements is complicated by the presence of a large excess of defective proviruses. This has been partially addressed by the intact proviral DNA assay (IPDA) [27], which relies on primers that are widely separated in the proviral genome and therefore enrich for sequences arising from intact genomes compared to the majority of deletions seen the in defective proviral population, which are clustered in either the 3′ or 5′ halves of the genome. A complementary method is to use PCR to measure the levels of inducible viral RNA [14, 28]. One of the most refined of these types of assays is the Tat/Rev Induced limiting dilution assay (TILDA), which uses limiting dilution to measure the frequency of CD4^+^ T cells producing HIV-1 multiply spliced (ms) Tat/Rev RNA upon mitogenic stimulation [29]. Since unspliced HIV-1 RNAs are frequently detected in latently infected cells in the absence of viral production, HIV-1 msRNAs (tat/rev) more accurately reflect active viral production [30, 31]. This assay represents an advance over Q-VOA but still fails to provide accurate quantitation, overcome sampling errors, and provide an adequate dynamic range to compare the efficiencies of various latency reversing agents. The EDITS assay is a PCR-based measuring mature spliced *env* mRNA in cells that also express functional Tat and Rev [32]. Sequence analysis shows that more than 98% of defective proviruses are unable to generate mature *env* mRNA [33]. Consequently, most cells expressing *env* mRNA are also capable of producing replication-competent virus [34]. Recently, two other groups have shown that both singly splice *vpu/env* mRNA and multiply spliced *tat/rev* mRNA measurements correlate well with replication competent virus levels [35, 36]. EDITS has been previously used by our group to demonstrate increases in the HIV-1 reservoir during reproductive aging of women [37] and the impact of IL-15 superagonists on the reduction of HIV-1 reservoirs [38].

Our general understanding of HIV-1 pathogenesis and response to ART is strongly biased towards subtype B, the predominant HIV-1 subtype in North America and Western Europe [39, 40]. Not surprisingly, most HIV-1 reservoir studies have followed a similar path, being performed in developed countries targeting individuals infected with subtype B HIV-1 strains [8, 15]. With more than two-thirds of the total global number of PLWH in Africa, mainly infected with non-HIV-1 subtypes [41], there is a clear need to study the HIV-1 reservoir in this population, especially in LMICs with multiple prevalent subtypes [42–44]. A few studies have described differences in the HIV-1 reservoir in individuals infected with non-subtype B HIV-1 strains compared to subtype B-infected patients [45, 46], highlighting the need to better understand the role of the immune response, co-infections, and/or viral factors associated with different HIV-1 subtypes in the latent HIV-1 reservoir. In this study, we modified and validated our novel deep sequencing-based EDITS assay [32] for non-clade B viruses before using it to characterize the peripheral HIV-1 reservoir in PLWH in Uganda. We measured not only the size but the diversity of the HIV-1 reservoir by comparing cell-associated HIV-1 RNA and proviral DNA from Ugandan individuals infected with non-B subtype HIV-1 strains, with U.S. patients infected with subtype B virus. We also evaluated the response of non-B and B subtype HIV-1 reservoirs to different latency reversing agents. The results showed unexpectedly that non-B subtype HIV-1 infections resulted in significantly lower reservoir sizes than those seen in in subtype B HIV-1 infections in the US.

## Results

### Development of an EDITS assay that accurately quantifies latently HIV-1-infected cells with B and non-B subtypes

Multiple assays have been developed to quantify the HIV-1 reservoir [15, 18, 19], most of them to be performed in North America, Europe, and Australia, where subtype B HIV-1 strains are predominant [41]. Since the main goal of this study was to evaluate the HIV-1 reservoir in Uganda, it was important to test the ability of the modified EDITS assay to measure inducible cell-associated spliced HIV-1 *env* mRNA in individuals infected with more worldwide prevalent non-B HIV-1 strains. We therefore designed new primer sets that bind in highly conserved regions of the HIV-1 genome (http://www.hiv.lanl.gov/content/sequence/HIV/mainpage.html) and tested these in cells infected with 32 diverse HIV-1 group M strains, including: five subtype A, six subtype B, six subtype C, seven subtype D, four subtype F, one subtype G, one subtype H, and two circulating recombinant form AE (CRF01_AE), plus one HIV-1 group O, one HIV-2 strain and seven multidrug resistant subtype B HIV-1 isolates. As expected, amplicons of the correct sizes were obtained—and number of reads quantified by deep sequencing—from all HIV-1 group M isolates analyzed; however, the primers were not able to amplify the more divergent group O (HIV-1_SC-O_) and HIV-2_CBL-20_ strains (Additional file 2: Fig. S1). The specificity of the primers was further tested using nucleic acids from a series of RNA and DNA viruses (i.e., BKV, CMV, HSV-1, HSV-2, VZV, HBV, HCV, and EBV). No cross-reactivity was observed with any of these viruses, as all PCR reactions failed to generate any detectable amplicons (Additional file 2: Fig. S1).

The sensitivity of the nested PCR amplification step was evaluated initially by mixing DNA from the HIV-1 molecular clone pNL4-3 with DNA from a non-HIV plasmid (pUC19). Plasmid DNA was quantified and serial dilutions were used to prepare nine mixtures containing pNL4-3 at 0% to 100% in a background of pUC19, at a final concentration of 0.1 ng/ml. Using both standard and real-time PCR, the system was able to detect 10 fg of pNL4-3 DNA in a background of 0.1 ng/ml of non-HIV DNA total plasmid concentration. Similar results were obtained by standard and real-time PCR, as well as by quantifying the number of reads using deep sequencing (Additional file 3: Fig. S2A).

When evaluating the HIV-1 reservoir it is important to relate PCR values to estimates of the numbers of infected cell (“cell equivalents”). Fully activated cells produce 1000 to 2000 copies of spliced HIV-1 *env* mRNA per cell and this is relatively uniform due activation of the Tat protein that leads to highly efficient HIV-1 transcription. It is therefore possible to establish a calibration curve relating changes in PCR values to changes in input cell numbers. To establish an initial calibration curve, and determine the cellular limit of detection for the EDITS assay using the new primer sets we initially used ACH-2 cells, a cell line latently infected with a copy of HIV-1_LAV_ per cell [47], and MT-4, an HIV-negative human T cell line as a control [48]. Cells were counted and serial dilutions were used to prepare seven mixtures containing 0 to 1000 ACH-2 cells in a background of 1 × 10^6^ MT-4 cells. The cell mixtures were activated with 10 μg/ml of Concanavalin A and cell-associated spliced HIV-1 RNA quantified using EDITS as described in “Methods” section. We were able to detect a minimum of 10 HIV^+^ ACH-2 cells in a milieu of one million HIV^−^ MT-4 cells (0.001%). Equally important, RNA from the two DNase-treated negative controls, i.e., (i) zero ACH-2 cells mixture and (ii) one million ACH-2 cells lacking the reverse transcriptase enzyme in the One-Step RT-PCR step, failed to show any HIV-specific mapped read (Additional file 3: Fig. S2B).

We further refined the analytical sensitivity of the EDITS assay by mimicking the peripheral HIV-1 reservoir in the host using two Jurkat cell lines: Jurkat E4, a cell line latently infected with a single copy of HIV-1 per cell [49], and Jurkat E6-1, an HIV-negative human T cell lymphoblast [50]. Cells were quantified and serial dilutions were used to prepare ten mixtures containing 0 to 400 Jurkat E4 cells in a background of 1 × 10^6^ Jurkat E6-1 cells. Following cell activation with Concanavalin A, cell-associated spliced HIV-1 RNA was quantified using EDITS assay and proviral DNA assessed by deep sequencing. The EDITS assay was able to accurately detect as low as eight HIV^+^ cells in one million HIV^−^ cells (*r* = 0.989, *p* < 0.0001, Pearson’s coefficient correlation), while the proviral DNA deep sequencing assay detected a minimum of 16 HIV^+^ cells (*r* = 0.997, *p* < 0.0001, Pearson’s coefficient correlation, Additional file 4: Fig. S3).

Finally, we validated the ability of the EDITS assay to quantify the peripheral HIV-1 reservoir by analyzing memory T cells from five ART-experienced, well-suppressed (plasma HIV-1 RNA load < 20 copies/ml) individuals infected with subtype B HIV-1 strains (Cleveland, OH) with (i) the EDITS assay, (ii) proviral DNA deep sequencing assay, and (iii) dPCR test designed to quantif proviral DNA. As expected, all three assays were able to quantify the HIV-1 reservoir in these patients, showing good correlation between EDITS and dPCR (*r* = 0.969, *p* < 0.006, Pearson’s coefficient correlation), EDITS and proviral DNA (*r* = 0.973, *p* < 0.005), and proviral DNA and dPCR (*r* = 0.979, *p* < 0.003, Additional file 5: Fig. S4).

### Reduced peripheral HIV-1 reservoir in Ugandan patients compared to HIV-infected individuals in the U.S

For this cross-sectional study we recruited HIV-infected individuals living in Kampala, Uganda (n = 62) and Cleveland, Ohio, USA (n = 50). Overall, Ugandan patients were slightly younger than individuals from the U.S. (median, 43 vs 49 years, *p* = 0.009), more likely to be female (63% vs 16%, *p* < 0.001), and reported only heterosexual risk of HIV exposure, while U.S. patients reported more diverse risk factors (*p* < 0.001, Table 1). Although both groups of individuals had undetectable plasma HIV RNA loads (< 20 copies/ml) at the time of blood collection, compared with U.S. patients, Ugandans had lower CD4^+^ T-cell counts (median 772 vs 508, *p* < 0.0001, Table 1). ART history showed that a similar number of individuals in Uganda and in the U.S. had been exposed to ART regimens containing protease (25 vs 27) and/or nucleoside(tide) reverse transcriptase (51 vs 50) inhibitors; however, more Ugandans received non-nucleoside reverse transcriptase inhibitors than U.S. patients (48 vs 35), while the opposite was true for integrase inhibitors (14 vs 28) and the entry inhibitor maraviroc (0 vs 3, p = 0.011, Table 1). Also, U.S. patients had a longer history of antiretroviral treatment, some starting suboptimal ART in the early 1990s. As expected, U.S. patients were exclusively infected with HIV-1 subtype B strains (n = 50), while individuals in Uganda were infected with HIV-1 subtypes A1 (n = 19), C (n = 3), and D (n = 40, Table 1).

**Table 1:** Demographic, clinical and virological characteristics

Characteristic	JCRC—Uganda (n = 62)	SIU—USA (n = 50)	*p* value
Median age (IQR)^a^	43 (33–50)	49 (40–57)	0.009
No. males (%)^b^	23 (37)	42 (84)	<0.0001
Exposure risk factor (#)^c^			
Heterosexual	62	6	
MSM	0	35	<0.0001
IVDU	0	5	
MTCT	0	4	
Median HIV-1 RNA (IQR)—c/ml^d^	< 20 (20–20)	<20 (20–20)	0.36
Median CD4^+^ T cells (IQR) —cell/mm^3e^	508 (399–757)	772 (594–981)	<0.0001
HIV–1 Subtype^f^ (#)	A (19), C (3), D (40)	B (50)	<0.0001
cART regimen —No. patients (mean, range number of drugs)^g^			
PI	25 (0.7, 0–2)	27 (1.4, 0–5)	0.011
NRTI	51 (1.7, 0–2)	50 (3.7, 2–7)	
NNRTI	48 (0.8, 0–1)	35 (0.9, 0–3)	
INSTI	14 (0.2, 0–1)	28 (0.7, 0–2)	
EI	0 (0, 0–0)	3 (0.1, 0–1)	

^a^Median age at the time of sampling; IQR, interquartile range

^b^Number and percentage of male patients

^c^Most likely mode of HIV-1 transmission: heterosexual; MSM, men who have sex with men; IVDU, intravenous drug user; MTCT, mother-to-child transmission; n.d., not determined

^d^Median HIV-1 RNA plasma load and IQR at the time the blood sample was obtained

^e^Median CD4^+^ T-cell count (cells/mm^3^) and IQR at the time the blood sample was obtained

^f^HIV-1 subtype based on phylogenetic analysis of patient-derived *vpu* sequences (see “Methods” section for details) and confirmed with both the DEEPGEN™ Software Tool Suite [51] and COMET HIV-1[52]

^g^Number of patients treated with combination antiretroviral therapy (cART), and mean number of antiretroviral drugs used per patient (*PI* protease inhibitors, *NRTI* nucleoside reverse transcriptase inhibitors, *NNRTI* non-nucleoside reverse transcriptase inhibitors, *INSTI* integrase strand transfer inhibitors; entry inhibitors)

*P* values based on Wilcoxon test (age), Fisher’s exact test (HIV-1 subtype and cART)

**Fig. 1:**
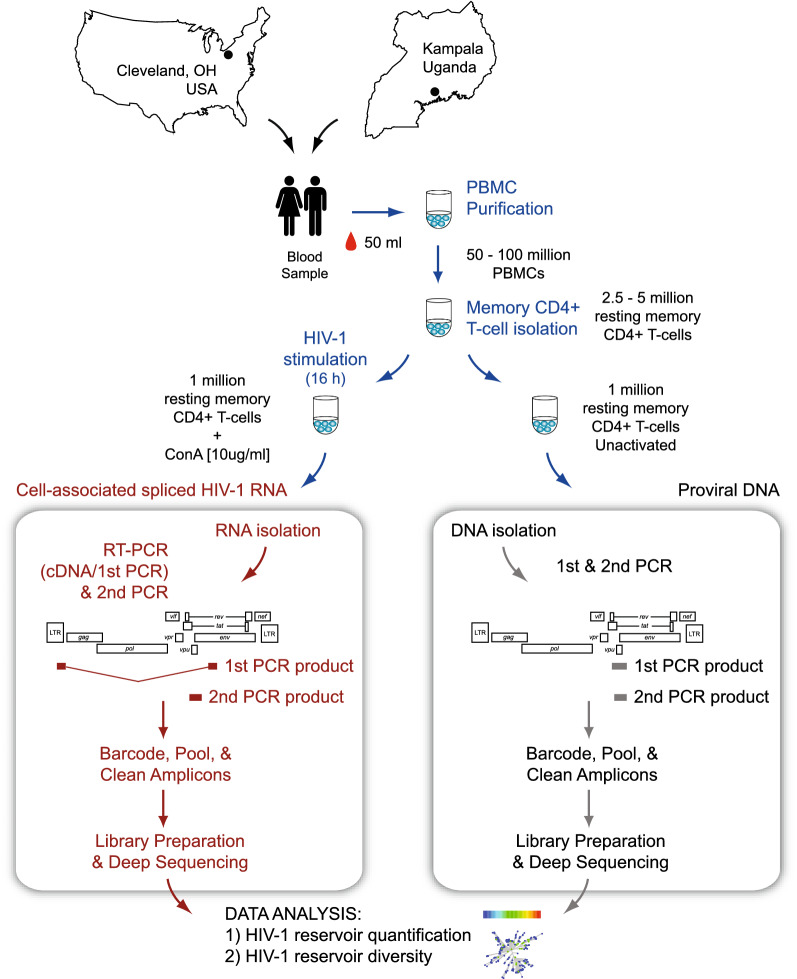
Overview of the EDITS assay. Blood samples were obtained from HIV-infected individuals in Cleveland, OH, USA and Kampala, Uganda. Peripheral blood mononuclear cells (PBMC) were isolated and CD4^+^ memory T cells purified, counted and purity verified by flow cytometry. One million CD4^+^ memory T cells were induced for 16 h with 10 μg/ml of the mitogen Concanavalin A (cell-associated spliced HIV-1 RNA). Total RNA was purified and used as template in a One-Step RT-PCR (external), with primers designed to bind to either side of the HIV-1 Env RNA splice junction, yielding a product of approximately 1.9 kb from the spliced HIV-1 *env* mRNA. A nested PCR amplification using barcoded primers produced a 369 bp fragment corresponding to *vpu/env* (HIV-1_HXB2_ position 6026 to 6394), which was purified, quantified, and deep sequenced (MiSeq Illumina). Reads were analyzed using the DEEPGEN™ Software Tool Suite [51] and converted into the equivalent number of cells harboring HIV-1 per 10^6^ cells using a standard curve as described [32]. The second aliquots of one million CD4^+^ memory T cells incubated in cell medium alone were used to isolate DNA. External PCR reactions amplified a 584 bp fragment, which was used as template for the nested PCR reaction, library preparation, deep sequencing, and bioinformatics analysis as described for the EDITS assay

**Fig. 2:**
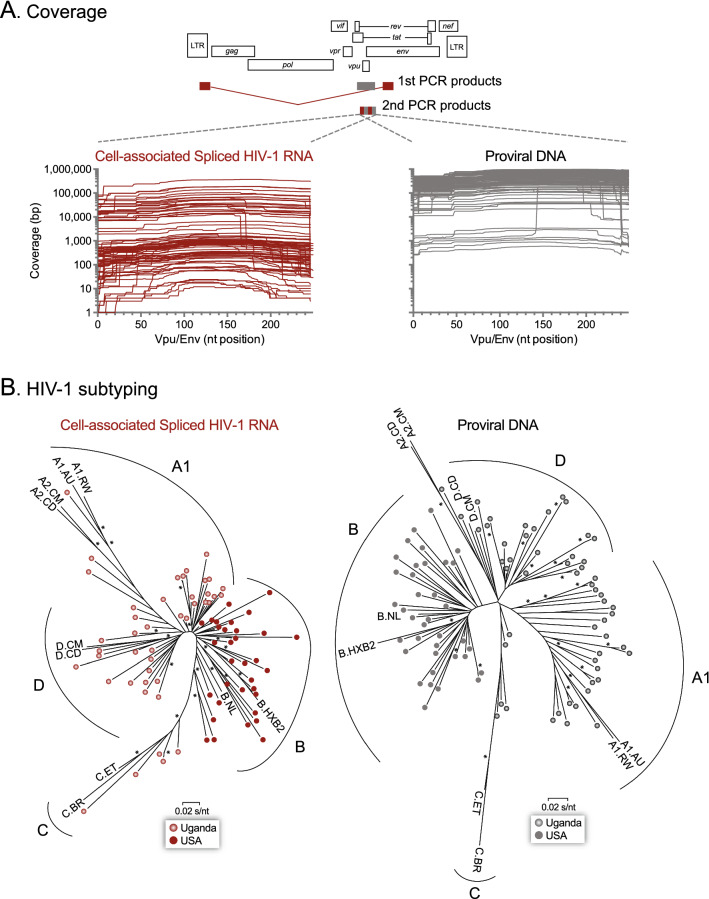
**A** Coverage, i.e., number of reads per nucleotide position, obtained by deep sequencing of cell-associated spliced HIV-1 RNA (EDITS assays) and proviral DNA from all Ugandan (n = 62) and U.S. (n = 50) HIV-infected individuals. The position relative to the HIV-1 genome of the vpu/env amplicon amplified in the nested (2nd) PCR reaction is indicated. **B** Neighbor-joining phylogenetic trees were constructed using the HIV-1 vpu/env consensus sequences generated for each patient-derived virus from deep sequencing reads using DEEPGEN™ Software Tool Suite [51], and rooted using the HIV-1_HXB2_ sequence (GenBank Accession Number AF033819). Nucleotide sequences from ten HIV-1 strains, two from each one of the HIV-1 subtypes more prevalent in Uganda and/or in the U.S., were used to subtype the patient-derived HIV-1 consensus sequences (**A1.AU**.03.PS1044_Day0.DQ676872, **A1.RW**.92.92RW008.AB253421, **A2.CD**.97.97CDKTB48.AF286238, **A2.CM**.01.01CM_1445MV.GU201516, **B.HXB2**_LAI_IIIB_BRU.K03455, **B.NL**.00.671_00T36.AY423387, **C.BR**.92.BR025_d.U52953, **C.ET**.86.ETH2220.U46016, **D.CD**.83.ELI.K03454, and **D.CM**.01.01CM_4412HAL.AY371157). HIV-1 subtype-specific clusters are depicted. Bootstrap resampling (1000 data sets) of the multiple alignment tested the statistical robustness of the tree, with percentage values above 75% indicated by an asterisk. s/site, substitutions per nucleotide site

Blood samples from each group of individuals were initially processed in their respective sites (Uganda and USA), following exactly the same protocols and using the same reagents (Fig. 1). An average of 87 million PBMCs were obtained from each individual (range 54 to 188 million), with no difference between Ugandan and U.S. patients (median 79 vs 82, *p* = 0.081). Memory CD4^+^ T-cells (mean 17.4 million) were isolated and purity determined (mean 96.7%, range 91.1% to 99.7%) before being activated and analyzed with the EDITS and proviral DNA assays (external or 1st PCR products and proviral DNA from Uganda patients were sent to CWRU for further analysis, Fig. 1). All 112 patient-derived samples, including the respective standard curves, were combined and analyzed in seven deep sequencing runs (mean 83 samples per deep sequencing run, range 47 to 96, Additional file 6: Fig. S5). Overall, deep sequencing metrics were similar among the different runs, including cluster density (mean 1,066 × 10^3^/mm^2^, range 761 to 1,378), cluster passing filter (mean 93.3%, range 88.3% to 96.5%), error rate (mean 2.46%, range 2.09% to 2.9%), and total reads passing filter (mean 51 × 10^6^ reads, 35 to 65 million, Additional file 6: Fig. S5). As expected, sequencing coverage (i.e., average number of reads that mapped to the sample-specific consensus reference) varied with each sample, ranging from 1 to 371,003 bp for cell-associated spliced HIV-1 RNA and from 252 to 985,303 bp for proviral DNA (Fig. 2A). Consensus sequences from cell-associated spliced HIV-1 RNA and proviral DNA samples were used to construct phylogenetic trees to confirm the HIV-1 subtype initially determined using the DEEPGEN™ Software Tool Suite [51] and COMET HIV-1 [52] (Fig. 2B). All viruses from both cohorts were CCR5-tropic viruses.

**Fig. 3:**
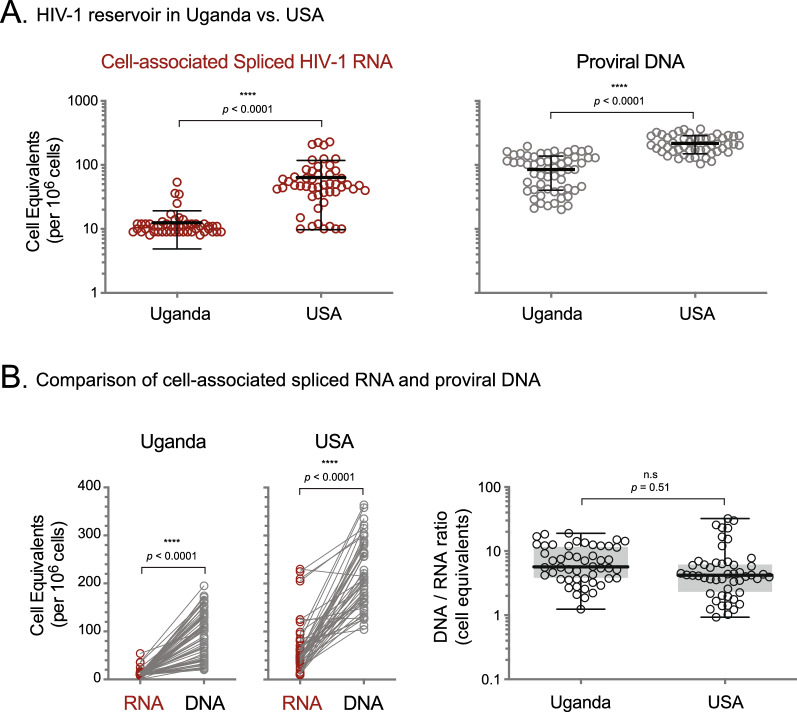
Quantification of the peripheral HIV-1 reservoir in patients from Uganda and USA. **A** The size of the inducible HIV-1 reservoir was determined using memory CD4^+^ T cells from Ugandan (n = 62) and U.S. (n = 50) patients by measuring cell-associated spliced HIV-1 RNA (EDITS assay). Proviral DNA estimated the number of cells carrying fully or partially activated proviruses. Unpaired t test was used to compare the reservoir size (number of cell equivalents per one million cells) between both cohort of patients. ****p < 0.0001. Median cell equivalents/million cells and interquartile range are depicted. **B** Comparison of the size of the inducible reservoir (RNA, cell-associated spliced HIV-1 RNA) and the reservoir estimated by quantifying proviral DNA (DNA) in each Ugandan and U.S. patient, as well as the ratio of the size of the peripheral HIV-1 reservoir determined by measuring proviral DNA vs cell-associated spliced RNA (DNA/RNA). Unpaired t test was used to compare the DNA/RNA ratio between Ugandan and U.S. group of patients. ****p < 0.0001; n.s., not significant

The size of the peripheral HIV-1 reservoir in Ugandan and U.S. patients was initially determined by measuring cell-associated spliced HIV-1 RNA using the EDITS assay and converting the number of reads (show in Fig. 2) to cell numbers based on a calibration curve, as described above (“cell equivalents”) (Additional file 4: Fig. S3–Additional file 7: Fig. S6). Interestingly, Ugandans had a fourfold smaller inducible HIV-1 reservoir than the one calculated for the patients in the U.S. (median 11 vs 48 cell equivalents/million cells, p < 0.0001, unpaired t test, Fig. 3A). A less marked, but still significant, two-fold difference was observed when the peripheral HIV-1 reservoir was quantified based on proviral DNA (median 88 vs 206 cell equivalents/million cells, p < 0.0001, unpaired t test, Fig. 3A). As expected, in both groups of patients the number of latently HIV-infected cells in the periphery was higher when we used proviral DNA vs inducible cell-associated spliced HIV-1 RNA (median 88 vs 11 cell equivalents/million cells, *p* < 0.0001 in Uganda and 206 vs 48 cell equivalents/million cells, *p* < 0.0001 in the U.S.). However, the proviral DNA/cell-associated spliced HIV-1 RNA ratio was similar in Ugandan and U.S. HIV-infected individuals (median 5.6 vs 4.2, *p* = 0.51 unpaired t test, Fig. 3B).

Since the initial steps of the EDITS assay, from blood collection to 1st PCR reaction (Fig. 1), were performed both in Uganda and in the U.S., we decided to verify that the number of resting memory CD4^+^ T-cells activated with Concanavalin A was exactly the same in both sites (although both laboratories followed the same strict standard operating procedures). For that, extracted DNA from 15 Ugandan and 25 U.S. patients, as well as one million cells from five points of the standard curve generated in USA, were used to PCR amplify a 197 bp fragment of the albumin gene and the amplicons were quantified (Qubit 2.0, Thermo Fisher Scientific). Not surprisingly, the number (one million) of patient-derived resting memory CD4^+^ T cells from Ugandan and U.S. individuals that were included in the EDITS assay was similar (median 11.8 vs 12.3 ng/µl of albumin, *p* = 0.163 unpaired t test) and no different than the one million cells used in the standard curves (median 12.3 ng/µl of albumin, Additional file 7: Fig. S6).

**Fig. 4:**
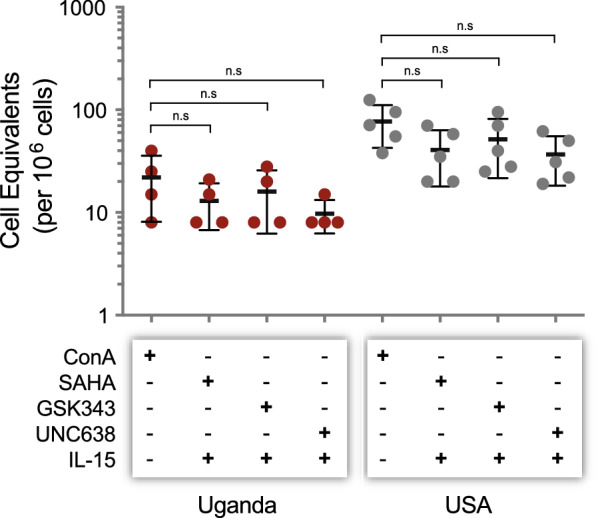
Effect of different agents to activate the inducible peripheral HIV-1 reservoir. Memory CD4^+^ T cells from Ugandan (n = 4) and U.S. (n = 5) patients were analyzed with the EDITS assay using, in addition to the standard 10 µg/ml of Concanavalin, a combination of IL-15 50 ng/ml plus SAHA 500 nM, IL-15 50 ng/ml plus GSK343 500 nM, or IL-15 50 ng/ml plus UNC638 500 nM. Unpaired t test was used to compare the reservoir size (number of cell equivalents per one million cells) induced by Concanavalin A vs the combination of agents to induce gene expression. Median cell equivalents/million cells and interquartile range are depicted. n.s., not significant

To rule out that variations in the induction efficiency of the peripheral HIV-1 reservoir with Concanavalin A could be playing a role in the different reservoir size in patients from Uganda and the U.S., we performed the EDITS assays as described in “Methods” section, but using a variety of different latency reversing conditions. This panel included combinations of IL-15 with HDAC inhibitors and methyltransferase inhibitors, which are known to reverse latency efficiently in B-clade samples (i.e., IL-15 50 ng/ml plus SAHA 500 nM, IL-15 50 ng/ml plus GSK343 500 nM, or IL-15 50 ng/ml plus UNC638 500 nM) [53–55]. As shown in Fig. 4, none of these combinations, which mediate epigenetic regulation of gene expression, showed a differential induction of the peripheral HIV-1 reservoir in patients from Uganda (median 11.5 vs 14 vs 8 cell equivalents/million cells, *p* > 0.891 one-way ANOVA) or the U.S. (median 35 vs 40 vs 31 cell equivalents/million cells, *p* < 0.952 one-way ANOVA). Moreover, induction of the peripheral HIV-1 reservoir with all three combinations of HIV-1 activation agents confirmed the smaller inducible HIV-1 reservoir in Ugandan individuals, compared to U.S patients, initially determined using Concanavalin A (Fig. 4).

Analysis of clinical and demographics characteristics showed that the size of the peripheral HIV-1 reservoir, measured by cell-associated HIV-1 RNA or proviral DNA, was not significantly associated with age, gender, exposure risk factor, plasma HIV-1 RNA load or CD4^+^ T cells at collection, or ART history (data not shown). The most evident correlation was with HIV-1 subtype, where all U.S. individuals were infected with subtype B while the Ugandan patients were infected with non-B HIV-1 strains (Table 1, Fig. 2B). Nonetheless, among Ugandans the inducible reservoir size was not associated with HIV-1 subtype (A, C, or D). The inducible HIV-1 reservoir size was not significantly different comparing male and female individuals within the same cohort of patients (median 10 vs 9 cell equivalents/million cells, *p* = 0.843 in Uganda and 48 vs 53 cell equivalents/million cells *p* = 0.974, unpaired t test in USA, Additional file 8: Fig. S7); however, comparing individuals from the same gender between both cohorts of patients resembled the overall smaller inducible HIV-1 peripheral reservoir described in Fig. 3A (i.e., male UG vs USA, median 10 vs 48 cell equivalents/million cells, *p* < 0.0001 and female UG vs USA, 9 vs 53 cell equivalents/million cells *p* = 0.035, unpaired t test, Additional file 8: Fig. S7). Similar results were obtained when the peripheral HIV-1 reservoir was quantified using proviral DNA (Additional file 8: Fig. S7). The proviral DNA pool in U.S. men was higher than women in this cohort, which probably reflects a higher number of defective proviruses that accumulated during suboptimal therapy, as typically seen in male HIV-infected individuals in the U.S.

### Peripheral HIV-1 reservoir in Ugandan patients is more diverse than in HIV-infected individuals from the U.S

**Fig. 5:**
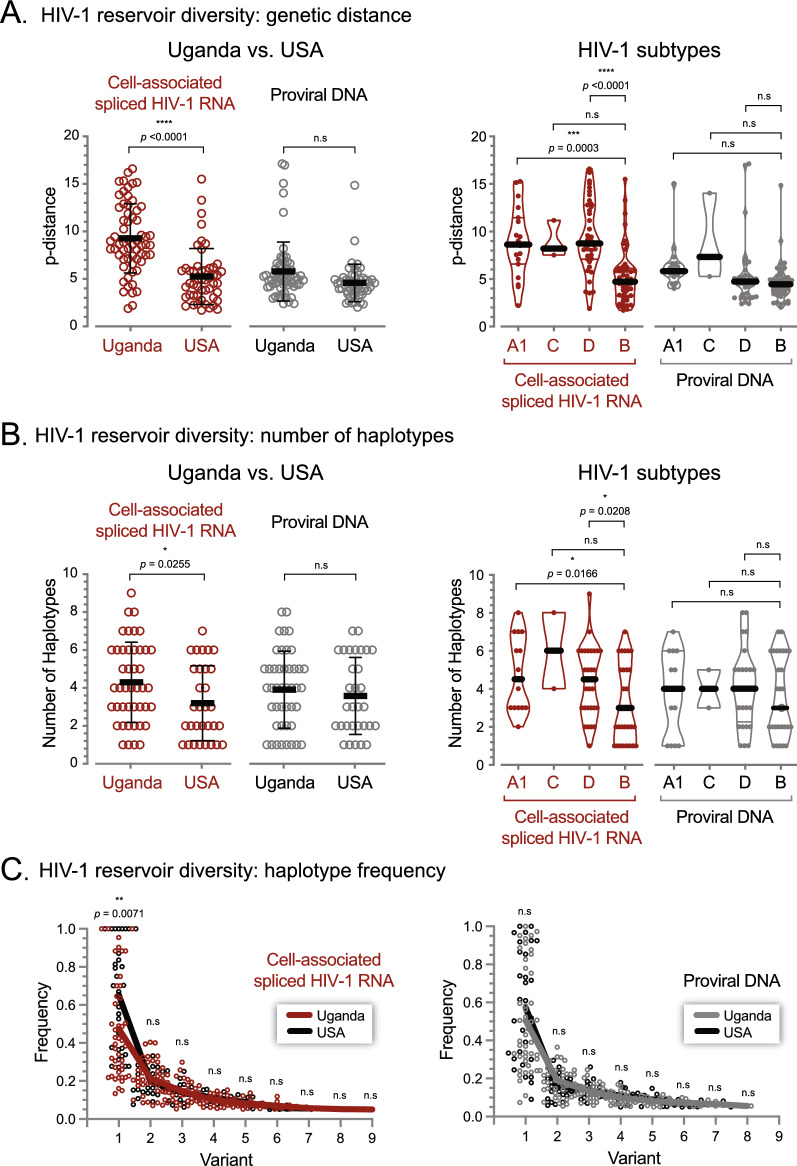
Genetic diversity of the peripheral HIV-1 reservoir in patients from Uganda and the U.S. **A** Intra-patient HIV-1 diversity of inducible cell-associated spliced HIV-1 RNA and proviral DNA in resting memory CD4^+^ T cells based on the p-distance model [56]. Unpaired t test was used to assess the statistical significance between the reservoir diversity in both cohorts of patients and between individuals infected with subtype B (U.S.) and non-B (Uganda) HIV-1 subtypes. Median substitutions/site and interquartile range are depicted. ***p < 0.001, ****p < 0.0001, n.s., not significant. **B** Genetic diversity of the inducible cell-associated spliced HIV-1 RNA and proviral DNA based on the number of viral haplotypes using CliqueSNV (103). Unpaired t test was used to compare the number of unique HIV-1 variants (viral haplotypes) between the reservoir diversity in both cohorts of patients and between individuals infected with subtype B (U.S.) and non-B (Uganda) HIV-1 subtypes. Median substitutions/site and interquartile range are depicted. *p < 0.05, n.s., not significant. **C** Comparison of the frequency of viral haplotypes in resting memory CD4^+^ T cells from Ugandan and U.S. patients. Each dot represents the frequency (proportional contribution) of the viral haplotype within the HIV-1 population when present as only one variant in the reservoir (1.0) or part of two or up to a maximum of nine unique sequences (> 0 to < 1) within each sample. Unpaired t test was used to compare the frequency of each viral haplotype present as one or more unique HIV-1 variant in each sample between both cohorts of patients. **p < 0.01, n.s., not significant

The EDITS assay not only quantifies the size of the peripheral reservoir by measuring inducible cell-associated spliced HIV-1 RNA but also, because sequences are obtained, allows assessment of the genetic diversity of the HIV-1 reservoir. We used two different bioinformatics approaches to estimate the HIV-1 quasispecies diversity in both inducible cell-associated spliced HIV-1 RNA and proviral DNA in resting memory CD4^+^ T cells from Ugandan and U.S. patients. First, we used the myriad of deep sequencing reads to calculate intra-patient HIV-1 diversity based on the p-distance model [56]. Interestingly, the cell-associated spliced HIV-1 RNA reservoir was more diverse in Ugandan patients than in HIV-infected individuals from the U.S. (median 8.6 vs 4.7 substitutions/site, *p* < 0.0001 unpaired t test); however, no significant difference was observed between both groups of patients when comparing proviral DNA diversity (Fig. 5A). Similar results were obtained by comparing the number of viral haplotypes in the peripheral HIV-1 reservoir determined by cell-associated spliced HIV-1 RNA, i.e., the reservoir from Ugandan patients had more unique HIV-1 variants than latently infected cells in U.S. patients (mean 4.3 vs 3.1, p = 0.025, unpaired t test), while no significant difference in the number of viral haplotypes was observed comparing proviral DNA diversity (Fig. 5B). The overall higher cell-associated spliced HIV-1 RNA diversity in the reservoir in Ugandan patients was confirmed when both p-distance and viral haplotypes were evaluated by subtype, that is, individuals infected with subtype A or D HIV-1 strains had more diverse peripheral reservoirs than patients infected with subtype B viruses (Fig. 5A and B). As expected, the diversity of the reservoir, based on cell-associated spliced HIV-1 RNA, also correlated with the frequency of unique variants within the viral population. In general, Ugandan patients had peripheral reservoirs with more than one viral haplotype, while more U.S. patients had reservoirs with only one unique HIV-1 variant (mean frequency 0.65 vs 0.47, *p* = 0.0071, unpaired t test), which again was not the case when diversity was determined using proviral DNA (Fig. 5C). Finally, the only parameter—other than subtype B vs non-B HIV-1 subtype—that was associated with inducible reservoir size quantified by cell-associated spliced HIV-1 RNA was HIV-1 RNA diversity (*p* = 0.0001 and *p* = 0.0003 in Ugandan and U.S. patients, respectively, Fisher’s exact test) but not when the size and diversity of the HIV-1 reservoir was determined by proviral DNA.

## Discussion

### Development of accurate measurements of the HIV-1 reservoir from non-subtype B infected patients

Combination antiretroviral therapy can durably suppress HIV-1 replication [57], effectively reducing this viral infection to a chronic disease; however, therapy must be taken continuously for life to prevent viral rebound. A series of strategies are being investigated to purge the HIV-1 reservoir, so a combination of the immune response and ART could effectively remove any vestige of the virus from the human host [8]. A key step to reach this goal is the development of highly sensitive and accurate methodologies capable of quantifying the HIV-1 reservoir in PLWH across the globe. In this study, we validated our novel deep sequencing-based assay to quantify the peripheral HIV-1 reservoir by measuring basal and inducible cell-associated spliced HIV-1 RNA. The EDITS assay specifically evaluates the multiply spliced *env* mRNA that spans more than 70% of the proviral genome, strongly enriching for sequences that derive from full-length HIV-1 genomes [32]. We then used EDITS to compare the peripheral HIV-1 reservoir profile in individuals infected with subtype B and non-B HIV-1 strains, showing that Ugandan patients infected with non-B viruses have smaller (fourfold) but more diverse (two-fold) HIV-1 reservoirs compared to U.S. individuals infected with subtype B viruses.

A number of different methodologies for HIV-1 reservoir quantification have been developed over the last two decades [15, 19, 58], although there is no clear correlation among most assays used to quantify latently HIV-infected cells [26]. The main challenge for all these cell-based and/or molecular assays has been the very limited number of latently HIV-infected cells potentially harboring replication-competent virus [3, 8]. Cell (culture)-based methods such as Q-VOA [20], TILDA [29], and ultrasensitive p24 ELISAs measure replication-competent viruses but can be labor intensive and might under or overestimate the size of the HIV-1 reservoir [15]. PCR-based assays to detect proviral DNA—either standard, real-time, or digital PCR—are relatively easier to perform but are not able to readily distinguish between replication-competent and defective HIV-1 genomes. An improvement on this was the development of the IPDA assay, which is a digital droplet PCR measurement using sets of widely separated primers to enrich for intact proviral DNA sequences [27]. This is generally regarded as providing an estimate of the upper limit of potentially fully infectious proviruses in the sample. Deep sequencing-based tests, like VOA-UDSA [59], quantify both defective and potentially replication-competent viruses; however, because of inefficient sampling they may still underestimate the HIV-1 reservoir.

Perhaps the most accurate approach to quantify the true size of the HIV-1 reservoir is to measure cell-associated HIV-1 RNA following activation of CD4^+^ T cells [19]. Our EDITS assay borrows features from both cell-based and molecular techniques, collecting and enriching for peripheral CD4^+^ memory T cells, which are activated to induce HIV-1 transcription. RT-PCR and deep sequencing of multiply spliced mRNA is a good surrogate for not only the quantification of the peripheral HIV-1 reservoir but also determining the diversity of full-length HIV-1 genomes that could generate replication-competent viruses [32]. This has been substantiated by viral outgrowth experiments, which have shown that there is a strong correlation between HIV-infected cells carrying inducible spliced mRNAs and cells harboring replication competent viruses [34–36, 60]. However, EDITS and related assays can be regarded as providing a lower level estimate of the HIV-1 reservoir since proviral activation ex vivo can be inefficient and a subset of activated cells could be lost due to cytopathic effects during activation. Nonetheless, in a recent side by side comparison of EDITS and IPDA reservoir measurements performed as part of a clinical evaluation of the IL-15 superagonist, N-803, the values obtained by both methods were not significantly different. In this case, the EDITS assay showed a lower standard deviation than the IPDA assay perhaps due to the large number of RNA molecules present in individually activated cells [38].

The original version of the EDITS assay used barcoded primers to RT-PCR amplify multiply spliced *env* mRNA products, which were then sequenced in the Personal Genome Machine (PGM) or S5 instruments (ThermoFisher) [32]. Unfortunately, this process was not optimized for clinical testing of divergent HIV-1 isolates since the length of the barcoded primers could affect not only the sensitivity of the assay but also its specificity, including the ability to amplify all non-B HIV-1 subtypes. Moreover, the intrinsic limitation of the PGM or S5 systems to discern insertions and deletions (indels) [61, 62] could affect the evaluation of the HIV-1 reservoir diversity. Therefore, we designed universal (non-barcoded) primers that amplify all HIV-1 subtypes and modified the library preparation and deep sequencing approach to include the MiSeq (Illumina) instrument, minimizing potential issues with the analysis of indels. Similar to other methodologies that are able to quantify the HIV-1 reservoir from individuals infected with non-B subtypes [63–66], the RT-PCR amplification and deep sequencing steps of the EDITS assay worked with all HIV-1 group M viruses analyzed. More importantly, EDITS matched the analytical sensitivity of other cellular- and/or PCR-based assays [15, 18], accurately detecting as low as eight HIV^+^ cells per million cells upon stimulation.

### Ugandans have reduced HIV-1 reservoirs with higher genetic diversity than U.S. patients

While our general knowledge of the HIV-1 latent reservoir, and potential cure strategies, has been mostly associated with subtype B HIV-1 infections [3, 8], a limited number of studies have analyzed the HIV-1 reservoir in individuals infected with non-B viruses. Similar to our findings, Prodger et al. [45] used Q-VOA to show that virally suppressed Ugandans had a threefold lower frequency of resting CD4^+^ T cells, compared with historical observations in U.S. patients. They were also able to demonstrate that whereas both sexes had similar total HIV-1 DNA levels, female patients had significantly fewer resting CD4^+^ T cells harboring replication-competent virus, as measured by viral outgrowth using QVOA, a feature that was not evident in our data due in part to the under- and over-representation of male individuals in our Ugandan and U.S. cohorts, respectively. A significantly larger HIV-1 reservoir was also described in a small number of patients infected with subtype B HIV-1 strains compared to individuals infected with subtype G or CRF01_AE viruses [46]. More recently, a combination of a TZM-bl cells-based assay (7.4 infectious units/10^6^ cells) and droplet digital PCR (129 HIV-1 copies/10^6^ cells) quantified the reservoir in adolescents infected with subtype C HIV-1 strains in Botswana [67], showing values similar to the median 11 and 88 cell equivalents/million cells we determined in Ugandan patients using cell-associated RNA and proviral DNA, respectively.

There are several different, and not mutually exclusive hypotheses that could explain the reduced reservoirs sizes seen in the Uganda patients. First, the HIV-1 subtypes themselves could be playing a role in the establishment and size of the latent HIV-1 reservoir. Each HIV-1 subtype has a specific LTR promoter configuration and minor changes in transcription factor binding sites could impact transcriptional activity [68]. For example, it has been long documented that subtype C viruses have additional NF-κB sites and higher replication rates than clade B viruses [69], suggesting that they may also be more responsive to T-cell activation and, thus, reduced HIV-1 reservoirs in the presence of ART. Also, subtype B HIV-1 *nef* sequences seems to have higher CD4 and HLA downregulation activities than the rest of the group M HIV-1 subtypes [70], perhaps contributing to the establishment of larger HIV-1 reservoir in individuals infected with subtype B HIV-1 strains [46].

An alternative explanation for the difference in the size of the peripheral HIV-1 reservoir in non-B HIV-infected patients relates to the geographic distribution of these non-B viruses, highly prevalent in LMICs [41]. In fact, a number of confounding factors associated with living in these countries could have an impact on the size of the latent HIV-1 reservoir. For example, higher coinfection prevalence compared to countries where subtype B viruses are common, could have an effect in the immunological milieu of HIV-infected individuals in LMICs [71]. It is possible that higher immune activation levels due to chronic infections could be constantly stimulating the HIV-1 reservoir, helping clearing latently HIV-infected cells in ART-experienced and well-suppressed (undetectable plasma HIV RNA load) individuals, as the patients described in this study. It is well known that infection with *Mycobacterium tuberculosis* can be associated with persistent systemic inflammation, which can reactivate and eventually decrease the latent HIV-1 reservoir [72, 73], while bacterial infection can also induce HIV-1 latency in macrophages [74]. On the other hand, individuals co-infected with cytomegalovirus [75, 76], hepatitis C virus [77], or herpes simplex virus type 2 [78] have been shown to have larger HIV-1 reservoirs, although quantified by measuring proviral DNA. Further studies, most likely in the form of clinical trials and/or longitudinal studies will be necessary to evaluate the role of acute and chronic coinfections in the latent HIV-1 reservoir. Interestingly, a study measuring proviral DNA by real-time PCR in a limited number of Brazilian individuals infected with subtype B, C, or F viruses showed no difference in the HIV-1 reservoir size [79]. Unlike our, and other studies comparing the reservoir in patients infected with different HIV-1 subtypes, these individuals were all recruited in the same geographic region, suggesting that factors other than HIV-1 subtype could be affecting the size and diversity of the peripheral HIV-1 reservoir.

Reservoir size and its complexity is strongly influenced by the clonal expansion of latently HIV-infected cells [80–87]. Early ART seems to limits HIV-1 reservoir size [8, 13], and the complexity of the reservoir has also been shown to be reduced [88, 89]. Treatment interruption studies suggest that a limited number of proviral DNA is responsible for new viremia outbursts following ART interruption [82, 83, 88, 89]. The results from our EDITS assay allows us to assess both the size of the peripheral HIV-1 reservoir by measuring the amount of inducible cell-associated spliced HIV-1 RNA and/or proviral DNA, and also the genetic diversity of the replication-competent and/or proviruses in the memory CD4^+^ T cells, which is a reflection of clonal diversity in the reservoir cell population. To improve our assessment of clonality we developed a proprietary pipeline to automatically calculate the size of the HIV-1 reservoir (RNA and/or DNA) as well as the p-distance, as a measurement of the intrapatient HIV-1 diversity (quasispecies), to determine the clonality of the peripheral HIV-1 reservoir. The results showed that although the number of latently HIV-infected cells in Ugandan patients is smaller than in U.S. individuals, the cell-associated spliced HIV-1 RNA reservoir was more diverse in people infected with non-B (Uganda) compared to subtype B (USA) HIV-1 strains. Our data is supported by a recent study showing that the HIV-1 reservoir in Zambian individuals infected with subtype C viruses recapitulated the extensive viral diversity accumulated during untreated HIV-1 infection [90]. Thus, it is possible that the same confounder factor(s), responsible for the elevated higher immune activation, could be constantly stimulating latently HIV-infected cells, favoring the clearing and re-seeding of the peripheral HIV-1 reservoir, increasing its diversity while simultaneously reducing its size.

This study has a few limitations. First, we focused exclusively on the peripheral HIV-1 reservoir, quantifying latently-infected CD4^+^ memory T cells [8, 10, 91, 92], which represent the major and well-defined pool of replication-competent latent HIV-1 [8]. Therefore, additional studies will be needed to evaluate the contribution from distant compartments in individuals infected with non-B HIV-1 subtypes, which cannot be sampled through peripheral blood. We also analyzed the HIV-1 reservoir from patients infected with subtype A or D, with a limited number of subtype C infections, representative of the HIV-1 subtype distribution in Uganda [93]. It will be interesting to expand our findings to the most worldwide prevalent subtype C viruses [3]. It was also difficult to collect a comprehensive clinical history for each one of the 112 patients included in this study. Further studies will need to record past and ongoing viral, bacterial, and/or fungal infections to evaluate their potential role on the latent HIV-1 reservoir and/or perform a longitudinal study where this vital information could be obtained. Finally, we used the well-characterized mitogen Concanavalin A to stimulate the CD4^+^ memory T cells, showing no difference in the induction of the peripheral HIV-1 reservoir compared to combinations of established LRAs such as SAHA, IL-15, GSK343, and UNC638. Other groups have used a number of agents to stimulate HIV-1 transcription, including anti-CD3/CD28 antibodies and multitude of LRAs [8, 94]; however, we chose this lectin for its cost-effectiveness ratio, aimed to implementing the EDITS assay not only in Uganda but in other LMICs.

## Conclusions

In this study we characterized and validated a new version of our deep sequencing-based EDITS assay to accurately measure the size and diversity of the peripheral HIV-1 reservoir. We used this innovative assay to show that Ugandans infected with non-B HIV-1 strains have a reduced but more diverse HIV-1 reservoir, compared to individuals infected with subtype B viruses living in the U.S. Geographic region, HIV-1 subtype, and inducible cell-associated spliced HIV-1 RNA diversity were the only parameters that correlated with the size of the inducible HIV-1 reservoir size, while responding similarly to activation with different latency reversing agents. Further, most likely longitudinal, studies are necessary to discern the contribution of viral (e.g., HIV-1 subtype) and/or host (e.g., co-infections and/or other co-morbidities) factors in the HIV-1 reservoir of people living with HIV in LMICs.

## Methods

### Plasmids, cells, viruses, and compounds

The HIV-1 NL4-3 infectious molecular clone (pNL4-3) was obtained from the NIH HIV Reagent Program, Division of AIDS, NIAID, NIH contributed by Dr. Malcolm Martin. Plasmid pUC19 was purchased from Thermo Fisher Scientific. The following cell lines were obtained through the NIH HIV Reagent Program, Division of AIDS, NIAID, NIH: MT-4 cells contributed by Dr. Douglas Richman, ACH-2 cells contributed by Dr. Thomas Folks, and Jurkat E6-1 cells contributed by Dr. Arthur Weiss. Latently HIV-1-infected Jurkat E4 cells were constructed by Dr. Jonathan Karn’s group [49]. All four cell lines were maintained in RPMI 1640/2 mM L-glutamine medium (Cellgro Mediatech, Manassas, VA) supplemented with 10% fetal bovine serum (FBS; Cellgro Mediatech), 10 mM N-2-hydroxyethylpiperazine-N-2-ethanesulfonic acid buffer (HEPES; Sigma-Aldrich, St Louis, MI), 100 U of penicillin/ml, and 100 μg of streptomycin/ml (Gibco Thermo Fisher Scientific, Waltham, MA). The following HIV-1 isolates were obtained from the NIH HIV Reagent Program, Division of AIDS, NIAID, NIH: HIV-1_A-92RW009(11–5)_, HIV-1_A-93RW020(11–12)_, HIV-1_B-92BR014(11–6)_, HIV-1_B-92TH593(11–9)_, HIV-1_B-US714(11–10)_, HIV-1_B-92US727(11–11)_, HIV-1_B-92US076(11–15)_, HIV-1_C-92BR025(11–7)_, HIV-1_D-92UG038(11–4)_, HIV-1_D-93UG065(11–8)_, HIV-1_D-94UG108(11–14)_, HIV-1_F-93BR020(11–13)_, HIV-1_AE-CMU02(11–16)_, and HIV-2_CBL-20_ or from Dr. Eric J. Arts’ laboratory at Western University, London, Canada: HIV-1_A-V115(09–153)_, HIV-1_A-V120(09–154)_, HIV-1_C-C20(09–155)_, HIV-1_C-C22(09–156)_, HIV-1_C-C18(09–157)_, HIV-1_C-C21(09–158)_, HIV-1_D-V122(09–159)_, HIV-1_D-V126(09–160)_, HIV-1_D-V89(09–161)_, HIV-1_F-CA20(09–163)_, HIV-1_F-V164(09–164)_. Additional reference material from different HIV-1 group M subtypes was obtained from SeraCare Life Sciences, Inc (Milford, MA): HIV-1_SC-A_, HIV-1_SC-B_, HIV-1_SC-C_, HIV-1_SC-D_, HIV-1_SC-F_, HIV-1_SC-G_, HIV-1_SC-H_, HIV-1_SC-AE_, and HIV-1_SC-O_. Plasma samples derived from individuals infected with multidrug-resistant HIV-1 variants were obtained from the AIDS Clinical Trials Unit (ACTU) at Case Western Reserve University (CWRU)/University Hospitals Cleveland Medical Center (UHCMC), with the understanding and written consent of each participant: HIV-1_B-13–140_, HIV-1_B-13–142_, HIV-1_B-13–145_, HIV-1_B-13–177_, HIV-1_B-14–342_, HIV-1_B-14–344_, and HIV-1_B-14–356_ as described below. Aliquots of additional RNA or DNA viruses were obtained from the Molecular Diagnostics or Medical Microbiology laboratories at UHCMC: BK virus (BKV), Cytomegalovirus (CMV), Herpes simplex virus 1 and 2 (HSV- 1 and HSV-20, and Varicella zoster virus (VZV) or the Division of Infectious Diseases, School of Medicine at CWRU: Hepatitis B virus (HBV), Hepatitis C virus (HCV), and Epstein-Barr virus (EBV). Finally, CD4^+^ memory T cells were treated with the following compounds: the mitogen Concanavalin A (Merck Sigma, Burlington, MA); the histone deacetylase inhibitor suberoylanilide hydroxamic acid (SAHA) [95] (Cayman Chemical, Ann Arbor, MI); the inhibitor of G9a and GLP histone-lysine methyltransferases, UNC638 [96] (Merck Sigma); GSK343, a potent and selective inhibitor of the histone-lysine N-methyltransferase enzyme EZH2 [97] (Merck Sigma); and recombinant human IL-15 (PeproTech, Rocky Hill, NJ).

### Study cohort

Study participants were recruited during routine patient monitoring from two well-characterized cohorts of HIV-infected individuals at the (i) Joint Clinical Research Centre (JCRC) in Kampala, Uganda and (ii) John T. Carey Special Immunology Unit (SIU) at UHCMC in Cleveland, Ohio, USA, between November 2016 and November 2018. Inclusion criteria included antiretroviral (ART)-experienced and well-suppressed (plasma HIV RNA load < 20 copies/ml) HIV-infected male or female individuals, ages 18 or older, with CD4^+^ T-cell counts equal or greater than 500 cells/mm^3^. Blood samples, clinical and demographics information was collected with the understanding and written consent of each participant, after the study was reviewed and approved by the JCRC (HS-3012) and UHCMC (01-98-55) institutional review boards.

### EDITS assay

A modified version of the Envelope Detection by Induced Transcription-based Sequencing (EDITS) assay [32], based on inducible cell-associated spliced HIV-1 RNA, was used to characterize the peripheral HIV-1 reservoir in patients from Kampala, Uganda and Cleveland, Ohio, USA:

i. *Blood sample collection and cell isolation* EDTA-treated venous blood samples (60 ml) were collected and plasma and peripheral blood mononuclear cells (PBMCs) were purified by Ficoll-Paque (Millipore Sigma) density gradient centrifugation as described [98]. CD4^+^ memory T cells (CD4^+^CD45RO^+^) were purified using the Memory CD4^+^ T cell isolation kit (Miltenyi BioteC, Bergisch Gladbach, Germany), counted and purity verified by flow cytometry using the following markers: CD4-FITC, CD8-APC-Cy7, CD45RA-APC, and CD45RO-PE (Becton Dickinson Biosciences, Franklin Lakes, NJ).
ii. *Cell activation* For each determination, 1 × 10^6^ CD4^+^ memory T cells were induced for 16 h with 10 μg/ml of the mitogen Concanavalin A (Millipore Sigma, Burlington, MA) in a 24-well plate at 37 °C, allowing maximal activation of the silent HIV-1 provirus [32]. A second aliquot of 1 × 10^6^ CD4^+^ memory T cells was incubated in cell medium alone to detect any cells carrying fully or partially activated proviruses, a strong indicator of poor suppression and recent new infections, as well as to analyze proviral DNA.
iii. *RNA extraction and RT-PCR amplification* Total RNA was purified (RNeasy Mini kit, Qiagen, Valencia, CA), treated with DNase I (QIAgen), and eluted in 20 µl of DNase/RNase-free water. The entire 20 µl sample was used as template in a One-Step RT-PCR (external) using Verso 1-step RT-qPCR kit (Thermo Fisher Scientific) and primers eF546 and eR7609 in a 50 µl reaction mixture with the following cycling conditions: one cycle at 50 °C for 15 min; one cycle at 95 °C for 15 min; and 35 cycles of 95 °C for 15 s, 60 °C for 30 s, and 72 °C for 60 s. Primers eF546 (5′-GCTTCAAGTAGTGTGTGCCC-3′, HIV-1_HXB2_ position 546) and eR7609 (5′-CTGAAGATCTCGGACTCATTGT-3′, position 7609) were designed to bind to either side of the HIV-1 Env RNA splice junction, yielding a product of approximately 1.9 kb from the spliced HIV-1 *env* mRNA, allowing the detection of late spliced HIV-1 transcripts without any potential proviral DNA amplification (limited by the more than 7000 nucleotide spacing of the primers on the HIV-1 genome [32]). Nested PCR reactions were carried out in 25-µl reactions containing 2 µl of the One-Step RT-PCR (external) product, 2 × Bestaq™ DNA polymerase mastermix (Applied Biological Materials, Richmond, Canada) and barcoded primers nF6026 (5′-Illumina_P5-adapter_Index_i5_CAAGCTTCTCTATCAAAGCAG-3′, position 6026) and nR6373 (5′-Illumina_P7_adapter_Index_i7_TCTGATGCACAAAATAGAGTGG-3′, position 6373) with the following cycling conditions: one cycle at 98˚C for 30 s and 35 cycles of 98 °C for 10 s, 60 °C for 10 s, and 72 °C for 10 s. A 369-fragment corresponding to *vpu/env* (HIV-1_HXB2_ position 6026 to 6394) was amplified and purified (QIAquick PCR purification kit).
iv. *Library preparation and deep sequencing* Dual barcodes (indices i5 or i7) and Illumina overhang adapter (P5 or P7) sequences were included in primers nF6026 and nR6363 as described in Additional file 1: Table S1. Following DNA purification (Agencourt AMPure XP, Beckman Coulter, Brea, CA), individual barcoded DNA samples were quantified (Qubit 2.0, Thermo Fisher Scientific) and pooled. Paired-end multiplexed libraries (up to 96 samples per deep sequencing run, including 5% PhiX as internal control) were diluted to 20 pM and denatured with NaOH prior to sequencing on the MiSeq system (Illumina, San Diego, CA) using the MiSeq reagent Kit v3 600 cycle (2 × 300 bp, Illumina).
v. *Bioinformatic analysis* Indexed reads were demultiplexed and filtered to remove short reads (< 80 bp), generating sample-specific fastq files using BaseSpace (Illumina). We used a modified version of DEEPGEN™ Software Tool Suite [51] to quantify the number of sample-specific mapped reads. Briefly, to minimize the amount of data loss during mapping due to the high HIV-1 sequence variability and to allow for inter-patient variation across the *vpu/env* region, sample-specific reference sequences were constructed for this HIV-1 genomic region (position 6026 to 6394 in the HIV-1_HXB2_ reference strain, GenBank Accession No. K03455). Mapping of reads from each sample occurred in three stages. First, a guide template (reference) for mapping was selected from the Los Alamos HIV Sequence Database (http://www.hiv.lanl.gov/content/sequence/HIV/mainpage.html) by comparing 100 randomly selected reads to the *vpu/env* region of all full-length sequences present within the HIV Sequence Database, resulting on the selection of a closely related and subtype-specific HIV-1 reference for each sample. This comparison was performed using a k-mer approach that rapidly identifies regions of similarity between any two sequences to select a guide sequence for mapping with minimal divergence from the read data, as such divergence is the primary cause of biased data loss [99]. Following the selection of the subtype-specific HIV-1 reference sequence, reads were mapped and aligned using the mapping algorithm previously described [100]. During mapping site indexes in relation to HIV-1_HXB2_ were also maintained. Next, to reduce diversity between reads and the reference template, a consensus was generated across each site of the reference sequence and reads re-mapped to this final sample-specific consensus template. Finally, the total number of mapped reads was converted into the equivalent number of cells harboring HIV-1 per 10^6^ cells using a standard curve as described [32]. These internal controls were prepared from activated CD4^+^ memory T cells (obtained from HIV seronegative individuals) infected with a replication-competent HIV-1-GFP virus, which were sorted by flow cytometry into single wells of a 96-well plate. Samples for the standard curves contained between 1 and 400 HIV-1-infected cells per well and 1 × 10^6^ uninfected cells. These samples were processed, barcoded, pooled, and deep sequenced together with the query samples as an internal control in each deep sequencing run.

### Proviral DNA assay

A modified version of the EDITS assay was used to quantify HIV-1 proviruses in patients from Kampala, Uganda and Cleveland, Ohio, USA. Briefly, the second aliquots of 1 × 10^6^ CD4^+^ memory T cells incubated in cell medium alone were used to isolate DNA (QIAamp DNA Mini kit, Qiagen), eluted in 20 µl of DNase/RNase-free water. External PCR reactions were carried out in a 50-µl mixture containing 5 µl of DNA, 0.2 mM dNTPs, 1 mM MgCl_2_, 5 units of Pfu Turbo DNA Polymerase (Stratagene, San Diego, CA) and primers Tat-1 and EN70 with the following cycling conditions: one cycle at 95 °C for 5 min; 35 cycles of 95 °C for 30 s, 55 °C for 30 s, and 72 °C for 60 s; and one cycle at 72 °C for 10 min. Primers Tat-1 (5′-CCTAAACTAGAGCCCTGGAACCATCC-3′, HIV-1_HXB2_ position 5846) and EN70 (5′-GGTACACAGGCATGTGTGGCCC-3′, position 6430) were designed to amplify a product of approximately 605 bp from the first exon of Tat to Env. Nested PCR reactions, library preparation, deep sequencing, and bioinformatics analysis (including standard curves) were carried as described above for the EDITS assay.

### HIV-1 subtyping and phylogenetic analysis

For each sample dataset, reads spanning nucleotide positions 6026 to 6394 (HIV-1_HXB2_, 369 bp) were extracted and truncated for phylogenetic analysis. Briefly, consensus sequences were generated for each patient-derived virus using DEEPGEN™ Software Tool Suite [51], as described above for the EDITS assay, aligned using ClustalW [101] and their phylogeny reconstructed using the Maximum Likelihood model with bootstrap as the variance estimation method (1000 replicates) as implemented within MEGA 6.1 [102]. HIV-1 subtype, initially predicted by phylogenetic analysis with HIV-1 references sequences obtained from Los Alamos HIV Sequence Database, was confirmed with both the DEEPGEN™ Software Tool Suite [51] and COMET HIV-1 (Luxembourg Institute of Health, https://comet.lih.lu) [52].

### Intra-patient HIV-1 genetic diversity

Quasispecies diversity in the peripheral HIV-1 reservoir was determined using two complementary methods based on reads spanning nucleotide positions 6026 to 6394 (HIV-1_HXB2_, 369 bp). First, intra-patient HIV-1 diversity was determined based on the p-distance model as described for deep sequencing [56]. Implemented in the DEEPGEN™ Software Tool Suite [51], p-distance measures the proportion of different nucleotide sites between two pair of sequences (reads). Next, the number and frequency of unique HIV-1 variants (viral haplotypes) within each clinical sample was determined using CliqueSNV [103], which accurately assemblies both majority and minority (i.e., frequencies as low as 0.1%) haplotypes and estimate their frequencies within the viral population.

### Real-time PCR amplification

Serial dilutions of pNL4-3 mixed with pUC19 were quantified by real-time PCR using primers nF6026 and nR6373. Each 25 µl real-time PCR reaction contained 5 µl DNA, 1 × PCR buffer (Invitrogen), 5 mM MgCl2, 0.2 mM dNTPs, 0.2 µM of each primer, SYBR Green dye diluted 1:2500 (Sigma), and 1.25 U Platinum Taq DNA polymerase (Thermo Fisher Scientific). PCR conditions consisted in one cycle at 98˚C for 30 s and 35 cycles of 98 °C for 10 s, 60 °C for 10 s, and 72 °C for 10 s. Real-time PCR amplification, data acquisition, and analysis were performed using the ABI, StepOnePlus Real-Time PCR System (Applied Biosystems, Walthman, MA).

### Cell input quantification

Albumin DNA was quantified to verify the cellular input level in the EDITS assay. Briefly, a 197 bp fragment was PCR amplified in 25-µl reactions containing one microliter of cellular DNA, 2 × Bestaq™ DNA polymerase mastermix (Applied Biological Materials) and primers Alb-S (5′-GCTGTCATCTCTTGTGGGCTGT-3′) and Alb-AS (5′-AAACTCATGGGAGCTGCTGGTT-3′) with PCR conditions as described [104]. Amplicons were purified (QIAquick PCR purification kit) and quantified (Qubit 2.0, Thermo Fisher Scientific).

### Digital PCR detection of proviruses

Cellular DNA from memory CD4^+^ T cells from HIV-infected individuals was isolated and quantified as described above. Proviral loads were measured using the QuantStudio® 3D Digital PCR System (ThermoFisher). Briefly, 1500 ng of cellular DNA (per chip) was mixed with Quantstudio® 3D digital PCR mastermix and an HIV-1-specific TaqMan Detection Assay (Life Technologies). PCR mixes were loaded onto QuantStudio® 3D Digital PCR 20 K Chips v2 using a QuantStudio® 3D Digital PCR Chip Loader. Sealed chips were cycled using a Dual Flat BlockGeneAMP® PCR System 9700 (Applied Biosystems) at 96 °C for 10 min, followed by 41 cycles of 60 °C for 2 min and 98 °C for 30 s, with a final extension at 60 °C for 2 min. Fluorescence was measured with a QuantStudio 3D Digital PCR Instrument and analyzed using Analysis Suite dPCR Cloud Software (Thermo Fisher Connect™). The number of human cells represented in each PCR mix was based on the concentration of cellular DNA and mass of the human diploid genome (6.6 × 10^−12^* g* DNA/cell).

### Statistical analyses

Descriptive results are expressed as median values, standard deviations, range, and confidence intervals. Pearson’s correlation coefficient was used to determine the strength of association between categorical variables. Group means were compared using a 2-sided *t*-test and group medians were compared using a 2-sided Wilcoxon-Mann–Whitney test. The Fisher’s exact test was used to test for significant association between categorical measures. Cell associated spliced HIV-1 RNA level and proviral DNA levels were log_10_ transformed and then modeled as a function of each predictor variable (by field site) using a univariate linear model (lm). The cutoff level for significance was set at 0.05 (*p* < 0.05). All analyses were performed with R version 3.6.2 (https://www.R-project.org) and plotted using GraphPad Prism v.6.0b (GraphPad Software, La Jolla, CA).

## Supplementary Information

**Additional file 1:** **Table S1.** Illumina overhang adapter (P5 or P7) and index (i5 or i7) forward and reverse primers used in the EDITS assay.

**Additional file 2: Figure S1.** Specificity of EDITS primers. **A** Thirty-two HIV-1 group M isolates, as well as eight RNA or DNA viruses (BKV, BK virus; CMV, Cytomegalovirus; HSV-1 and HSV-2, Herpes simplex virus 1 and 2; VZV, Varicella zoster virus; HBV, Hepatitis B virus; HCV, Hepatitis C virus; and EBV, Epstein-Barr virus) were used to RT-PCR amplify a vpu/env 369 bp fragment and used to construct a neighbor-joining phylogenetic tree as described in “Methods” section. HIV-1 subtype-specific clusters are depicted. Bootstrap resampling (1000 data sets) of the multiple alignments, with percentage values above 75% are indicated by an asterisk. s/nt, substitutions per nucleotide. **B** The same 369 bp amplicons were deep sequenced, analyzed and reads quantified using the DEEPGEN™ Software Tool Suite.

**Additional file 3: Figure S2.** Sensitivity of EDITS primers. **A** Nested PCR primers nF6026 and nR6773 were evaluated using a serial dilution of DNA from the HIV-1 molecular clone pNL4-3 (0% to 100%) in a background of DNA from the non-HIV plasmid pUC19, at a final concentration of 0.1 ng/ml. DNA mixtures were amplified using Standard and Real-time PCR. Amplicons from the standard PCR were also deep sequenced and vpu/env mapped reads quantified using the DEEPGEN™ Software Tool Suite. Mean mapped reads and standard deviation are depicted. **B** ACH-2, a cell line latently infected with a copy of HIV-1 per cell, and MT-4, an HIV-negative human T cell line, were quantified and serial dilutions used to prepare seven mixtures containing 0 to 1000 CH-2 cells in a background of one million MT-4cells. Cell mixtures were ctivated with 100 μg/ml of Concanavalin A and cell-associated spliced HIV-1 RNA quantified using EDITS as described in “Methods” section. Mean mapped reads and standard deviation are depicted.

**Additional file 4: Figure S3.** Analytical sensitivity of the EDITS assay. Jurkat E4, a cell line latently infected with a single copy of HIV-1 per cell, and Jurkat E6-1, an HIV-negative human T cell lymphoblast, were quantified and serial dilutions were used to prepare ten mixtures containing 0 to 400 Jurkat E4 cells in a background of one million Jurkat E6-1 cells in eight biological replicates. Cell mixtures were activated and cell-associated spliced HIV-1 RNA and proviral DNA quantified using EDITS assay as described in “Methods” section. Heatmaps indicate the number of HIV-1 vpu/end reads in each of the eight cell mixture replicates. Linear dynamic ranges and regression values (Pearson’s coefficient correlation) describing the relationship between mapped vpu/env reads and number of HIV positive cells in the mixtures are indicated. Median mapped reads and interquartile range are depicted.r, correlation coeficient; p, two-tailed p value.

**Additional file 5: Figure S4.** Validation of the EDITS assay. Memory CD4+ T cells from five ART-experienced, well-suppressed (plasma HIV-1 RNA load < 20 copies/ml) individuals infected with HIV-1 subtype B strains in Cleveland, OH were used to quantify the peripheral HIV-1 reservoir using the EDITS assay, proviral DNA deep sequencing assay, and a dPCR test designed to quantify proviral DNA as described in “Methods” section. Linear dynamic ranges and regression values (Pearson’s coefficient correlation) describing the relationship between the size of the HIV-1 reservoir quantified by each assay are indicated. r, correlation coeficient; p, two-tailed p value.

**Additional file 6: Figure S5.** Relevant metrics generated during each one of the deep sequencing runs corresponding to the EDITS assay and/or the proviral DNA test. Number of samples included in each specific MiSeq run. Clusters Passing Filter (Cluster PF) and Total Reads Passing Filters (Total Reads PF), represent the percentage of generated clusters and number of reads, respectively, that passed an internal quality filtering procedure used by Illumina. Cluster Density indicates the amount of clusters that were generated per flow cell surface area during the cluster generation stage. Error rate was calculated based on the PhiX control samples spiked in each deep sequencing run, showing the number of bases with a mismatch relative to the PhiX sequence. Median values and interquartile range are depicted.

**Additional file 7: Figure S6.** Quantification of number of cells used in the EDITS assay. Cellular DNA was extracted from one million memory CD4+ T cells purified from 15 Ugandan and 25 U.S. patients, as well as one million Jurkat E4 (HIV+)/Jurkat E^-1 (HIV-) cells from five points of the standard curve. One microlliter of cellular DNA was use to PCR amplify a 197 bp fragment of the cellular albumin gene. Amplicons were purified and quantified using Qubit 2.0 (Thermo Fisher Scientific). Unpaired t test was used to compare the concentration of albumin amplicon (ng/ul) between both cohort of patients and the cell mixture from the standard curve. Median values and interquartile range are depicted.

**Additional file 8: Figure S7.** Size of the inducible peripheral HIV-1 reservoir in male and female HIV-infected individuals from Uganda (n = 62) and the U.S. (n =50) estimated by measuring cell-associated spliced HIV-1 RNA (EDITS assay) and proviral DNA. Unpaired t test was used to compare the reservoir size (number of cell equivalents per one million cells) between the different groups of patients. *p < 0.05, **p <0.01, ***p <0.001, ****p <0.0001. Median cell equivalents/million cells and interquartile range are depicted. ♂ male; ♀ female.

## Data Availability

The datasets analyzed in this study are available from the corresponding author on reasonable request.

## Abbreviations

ART: Antiretroviral therapy
DNA: Deoxyribonucleic acid
EDITS: Envelope Detection by Induced Transcription-based Sequencing (EDITS) assay
ELISA: Enzyme-linked immunosorbent assay
HDACI: Histone deacetylase inhibitors
HIV-1: Human immunodeficiency virus type 1
IPDA: Intact proviral DNA assay
LMIC: Low- and middle-income countries
LRA: Latency reversal agents
PCR: Polymerase chain reaction
PGM: Personal Genome Machine
PLWH: People living with HIV
Q-VOA: Quantitative viral outgrowth assay
RNA: Ribonucleic acid
SAHA: Suberoylanilide hydroxamic acid
TILDA: Tat/Rev Induced limiting dilution assay
U.S.: United States
