# Supplementary material for: Reduced and highly diverse peripheral HIV-1 reservoir in virally suppressed patients infected with non-B HIV-1 strains in Uganda
Source: Retrovirology. 2022 Jan 15;19:1. doi: 10.1186/s12977-022-00587-3 (PMC8760765; doi:10.1186/s12977-022-00587-3)
Supplement: Supplementary file 1 — Additional file 1: Table S1. Illumina overhang adapter (P5 or P7) and index (i5 or i7) forward and reverse primers used in the EDITS assay. [file 12977_2022_587_MOESM1_ESM.pdf]

Supplementary Table 1. Illumina overhang adapter (P5 or P7) and index (i5 or i7) forward and reverse primers used in the EDITS assay

Index Name-5'	Illumina P5 Adapter Sequence	Index i5	nF6026 Forward Primer	Primer Name	Barcoded primer Sequence (5' - 3')
S501	AATGATACGGCGACCACCGA	TAGATCGC	caagcttctctatcaaagcag	S501_nF6026	AATGATACGGCGACCACCGATAGATCGCcaagcttctctatcaaagcag
S502	AATGATACGGCGACCACCGA	CTCTCTAT	caagcttctctatcaaagcag	S502_ nF6026	AATGATACGGCGACCACCGACTCTCTATcaagcttctctatcaaagcag
S503	AATGATACGGCGACCACCGA	TATCCTCT	caagcttctctatcaaagcag	S503_ nF6026	AATGATACGGCGACCACCGATATCCTCTcaagcttctctatcaaagcag
S504	AATGATACGGCGACCACCGA	AGAGTAGA	caagcttctctatcaaagcag	S504_ nF6026	AATGATACGGCGACCACCGAAGAGTAGAcaagcttctctatcaaagcag
S505	AATGATACGGCGACCACCGA	GTAAGGAG	caagcttctctatcaaagcag	S505_ nF6026	AATGATACGGCGACCACCGAGTAAGGAGcaagcttctctatcaaagcag
S506	AATGATACGGCGACCACCGA	ACTGCATA	caagcttctctatcaaagcag	S506_ nF6026	AATGATACGGCGACCACCGAACTGCATAcaagcttctctatcaaagcag
S507	AATGATACGGCGACCACCGA	AAGGAGTA	caagcttctctatcaaagcag	S507_ nF6026	AATGATACGGCGACCACCGAAAGGAGTAcaagcttctctatcaaagcag
S508	AATGATACGGCGACCACCGA	CTAAGCCT	caagcttctctatcaaagcag	S508_ nF6026	AATGATACGGCGACCACCGACTAAGCCTcaagcttctctatcaaagcag

Index Name-3'	Illumina P7 Adapter sequence	Index i7 *	nR6373 Reverse Primer	Primer Name	Barcoded primer Sequence (5' - 3')
N701	CAAGCAGAAGACGGCATACGAGAT	TAAGGCGA	tctgatgcacaaaatagagtgg	N701_nR6373	CAAGCAGAAGACGGCATACGAGATTAAGGCGAtctgatgcacaaaatagagtgg
N702	CAAGCAGAAGACGGCATACGAGAT	CGTACTAG	tctgatgcacaaaatagagtgg	N702_ nR6373	CAAGCAGAAGACGGCATACGAGATCGTACTAGtctgatgcacaaaatagagtgg
N703	CAAGCAGAAGACGGCATACGAGAT	AGGCAGAA	tctgatgcacaaaatagagtgg	N703_ nR6373	CAAGCAGAAGACGGCATACGAGATAGGCAGAAtctgatgcacaaaatagagtgg
N704	CAAGCAGAAGACGGCATACGAGAT	TCCTGAGC	tctgatgcacaaaatagagtgg	N704_ nR6373	CAAGCAGAAGACGGCATACGAGATTCCTGAGCtctgatgcacaaaatagagtgg
N705	CAAGCAGAAGACGGCATACGAGAT	GGACTCCT	tctgatgcacaaaatagagtgg	N705_ nR6373	CAAGCAGAAGACGGCATACGAGATGGACTCCTtctgatgcacaaaatagagtgg
N706	CAAGCAGAAGACGGCATACGAGAT	TAGGCATG	tctgatgcacaaaatagagtgg	N706_ nR6373	CAAGCAGAAGACGGCATACGAGATTAGGCATGtctgatgcacaaaatagagtgg
N707	CAAGCAGAAGACGGCATACGAGAT	CTCTCTAC	tctgatgcacaaaatagagtgg	N707_ nR6373	CAAGCAGAAGACGGCATACGAGATCTCTCTACtctgatgcacaaaatagagtgg
N708	CAAGCAGAAGACGGCATACGAGAT	CAGAGAGG	tctgatgcacaaaatagagtgg	N708_ nR6373	CAAGCAGAAGACGGCATACGAGATCAGAGAGGtctgatgcacaaaatagagtgg
N709	CAAGCAGAAGACGGCATACGAGAT	GCTACGCT	tctgatgcacaaaatagagtgg	N709_ nR6373	CAAGCAGAAGACGGCATACGAGATGCTACGCTtctgatgcacaaaatagagtgg
N710	CAAGCAGAAGACGGCATACGAGAT	CGAGGCTG	tctgatgcacaaaatagagtgg	N710_ nR6373	CAAGCAGAAGACGGCATACGAGATCGAGGCTGtctgatgcacaaaatagagtgg
N711	CAAGCAGAAGACGGCATACGAGAT	AAGAGGCA	tctgatgcacaaaatagagtgg	N711_ nR6373	CAAGCAGAAGACGGCATACGAGATAAGAGGCAtctgatgcacaaaatagagtgg
N712	CAAGCAGAAGACGGCATACGAGAT	GTAGAGGA	tctgatgcacaaaatagagtgg	N712_ nR6373	CAAGCAGAAGACGGCATACGAGATGTAGAGGAtctgatgcacaaaatagagtgg

Combinations of one of the 8 forward and 12 reverse primers allowed to multiplex up to 96 samples in a single MiSeq sequencing run
