# Supplementary material for: Reduced and highly diverse peripheral HIV-1 reservoir in virally suppressed patients infected with non-B HIV-1 strains in Uganda
Source: Retrovirology. 2022 Jan 15;19:1. doi: 10.1186/s12977-022-00587-3 (PMC8760765; doi:10.1186/s12977-022-00587-3)
Supplement: Supplementary file 2 — Additional file 2: Figure S1. Specificity of EDITS primers. A Thirty-two HIV-1 group M isolates, as well as eight RNA or DNA viruses (BKV, BK virus; CMV, Cytomegalovirus; HSV-1 and HSV-2, Herpes simplex virus 1 and 2; VZV, Varicella zoster virus; HBV, Hepatitis B virus; HCV, Hepatitis C virus; and EBV, Epstein-Barr virus) were used to RT-PCR amplify a vpu/env 369 bp fragment and used to construct a neighbor-joining phylogenetic tree as described in “Methods” section. HIV-1 subtype-specific clusters are depicted. Bootstrap resampling (1000 data sets) of the multiple alignments, with percentage values above 75% are indicated by an asterisk. s/nt, substitutions per nucleotide. B The same 369 bp amplicons were deep sequenced, analyzed and reads quantified using the DEEPGEN™ Software Tool Suite. [file 12977_2022_587_MOESM2_ESM.pdf]

A. RT-PCR amplification of multiple HIV-1 subtypes (amplicon yield)

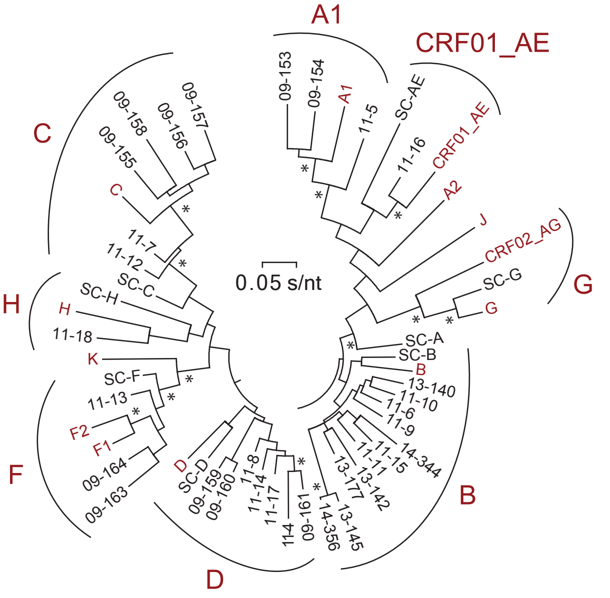

B. Deep sequencing-based quantification of multiple HIV-1 subtypes

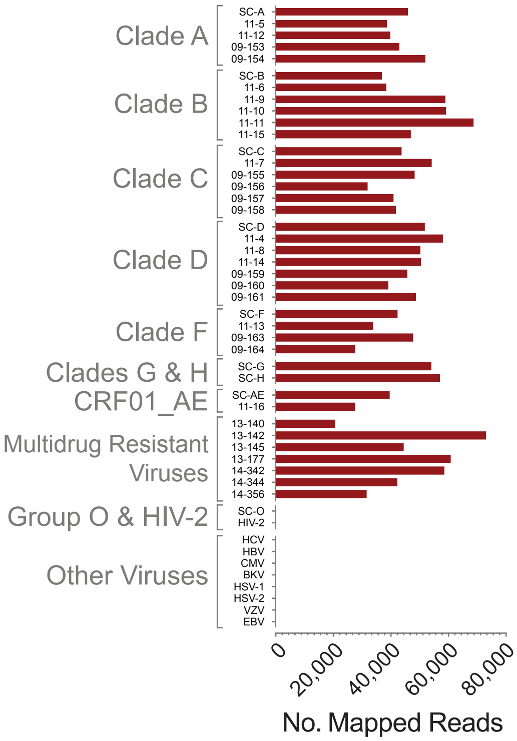

Supplementary Figure 1. Specificity of EDITS primers. (A) Thirty-two HIV-1 group M isolates, as well as eight RNA or DNA viruses (BKV, BK virus; CMV, Cytomegalovirus; HSV-1 and HSV-2, Herpes simplex virus 1 and 2; VZV, Varicella zoster virus; HBV, Hepatitis B virus; HCV, Hepatitis C virus; and EBV, Epstein-Barr virus) were used to RT-PCR amplify a vpu/env 369 bp fragment and used to construct a neighbor-joining phylogenetic tree as described in Materials & Methods. HIV-1 subtype-specific clusters are depicted. Bootstrap resampling (1,000 data sets) of the multiple alignments, with percentage values above 75% are indicated by an asterisk. s/nt, substitutions per nucleotide. (B) The same 369 bp amplicons were deep sequenced, analyzed and reads quantified using the DEEPGEN™ Software Tool Suite.
