# Supplementary material for: Reduced and highly diverse peripheral HIV-1 reservoir in virally suppressed patients infected with non-B HIV-1 strains in Uganda
Source: Retrovirology. 2022 Jan 15;19:1. doi: 10.1186/s12977-022-00587-3 (PMC8760765; doi:10.1186/s12977-022-00587-3)
Supplement: Supplementary file 3 — Additional file 3: Figure S2. Sensitivity of EDITS primers. A Nested PCR primers nF6026 and nR6773 were evaluated using a serial dilution of DNA from the HIV-1 molecular clone pNL4-3 (0% to 100%) in a background of DNA from the non-HIV plasmid pUC19, at a final concentration of 0.1 ng/ml. DNA mixtures were amplified using Standard and Real-time PCR. Amplicons from the standard PCR were also deep sequenced and vpu/env mapped reads quantified using the DEEPGEN™ Software Tool Suite. Mean mapped reads and standard deviation are depicted. B ACH-2, a cell line latently infected with a copy of HIV-1 per cell, and MT-4, an HIV-negative human T cell line, were quantified and serial dilutions used to prepare seven mixtures containing 0 to 1000 CH-2 cells in a background of one million MT-4cells. Cell mixtures were ctivated with 100 μg/ml of Concanavalin A and cell-associated spliced HIV-1 RNA quantified using EDITS as described in “Methods” section. Mean mapped reads and standard deviation are depicted. [file 12977_2022_587_MOESM3_ESM.pdf]

A. EDITS primers sensitivity (mixtures of plasmids)

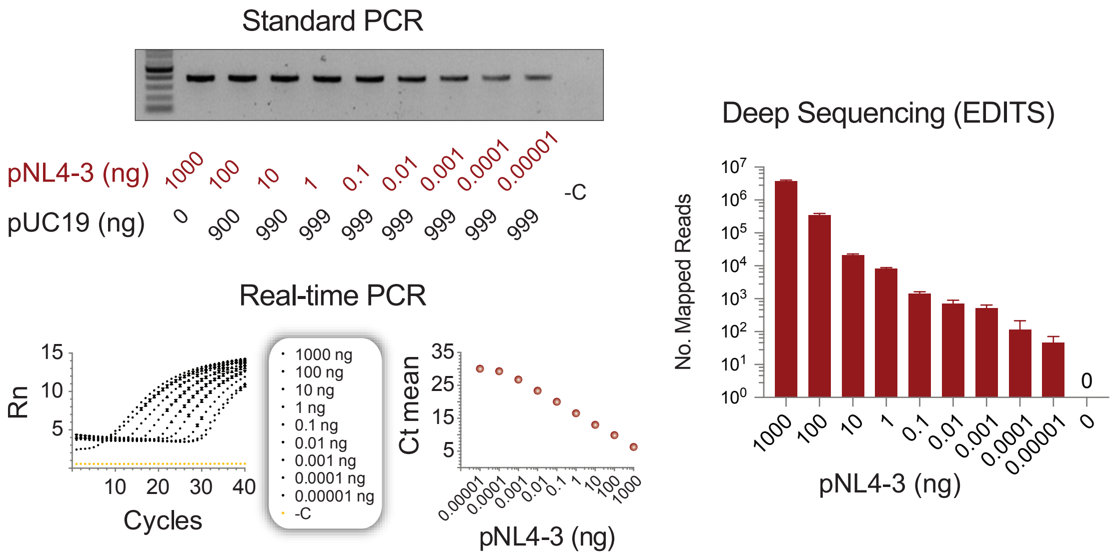

B. EDITS primers sensitivity (mixtures of cells)

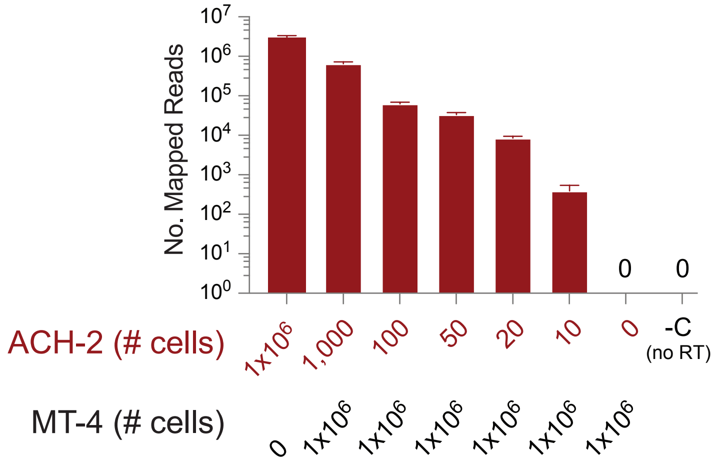

Supplementary Figure 2. Sensitivity of EDITS primers. (A) Nested PCR primers nF6026 and nR6773 were evaluated using a serial dilution of DNA from the HIV-1 molecular clone pNL4-3 (0% to 100%) in a background of DNA from the non-HIV plasmid pUC19, at a final concentration of 0.1 ng/ml. DNA mixtures were amplified using Standard and Real-time PCR. Amplicons from the standard PCR were also deep sequenced and vpu/env mapped reads quantified using the DEEPGEN™ Software Tool Suite. Mean mapped reads and standard deviation are depicted. (B) ACH-2, a cell line latently infected with a copy of HIV-1 per cell, and MT-4, an HIV-negative human T cell line, were quantified and serial dilutions used to prepare seven mixtures containing 0 to 1,000 ACH-2 cells in a background of one million MT-4cells. Cell mixtures were activated with 100 µg/ml of Concanavalin A and cell-associated spliced HIV-1 RNA quantified using EDITS as described in Materials & Methods. Mean mapped reads and standard deviation are depicted.
