# Supplementary material for: Reduced and highly diverse peripheral HIV-1 reservoir in virally suppressed patients infected with non-B HIV-1 strains in Uganda
Source: Retrovirology. 2022 Jan 15;19:1. doi: 10.1186/s12977-022-00587-3 (PMC8760765; doi:10.1186/s12977-022-00587-3)
Supplement: Supplementary file 4 — Additional file 4: Figure S3. Analytical sensitivity of the EDITS assay. Jurkat E4, a cell line latently infected with a single copy of HIV-1 per cell, and Jurkat E6-1, an HIV-negative human T cell lymphoblast, were quantified and serial dilutions were used to prepare ten mixtures containing 0 to 400 Jurkat E4 cells in a background of one million Jurkat E6-1 cells in eight biological replicates. Cell mixtures were activated and cell-associated spliced HIV-1 RNA and proviral DNA quantified using EDITS assay as described in “Methods” section. Heatmaps indicate the number of HIV-1 vpu/end reads in each of the eight cell mixture replicates. Linear dynamic ranges and regression values (Pearson’s coefficient correlation) describing the relationship between mapped vpu/env reads and number of HIV positive cells in the mixtures are indicated. Median mapped reads and interquartile range are depicted.r, correlation coeficient; p, two-tailed p value. [file 12977_2022_587_MOESM4_ESM.pdf]

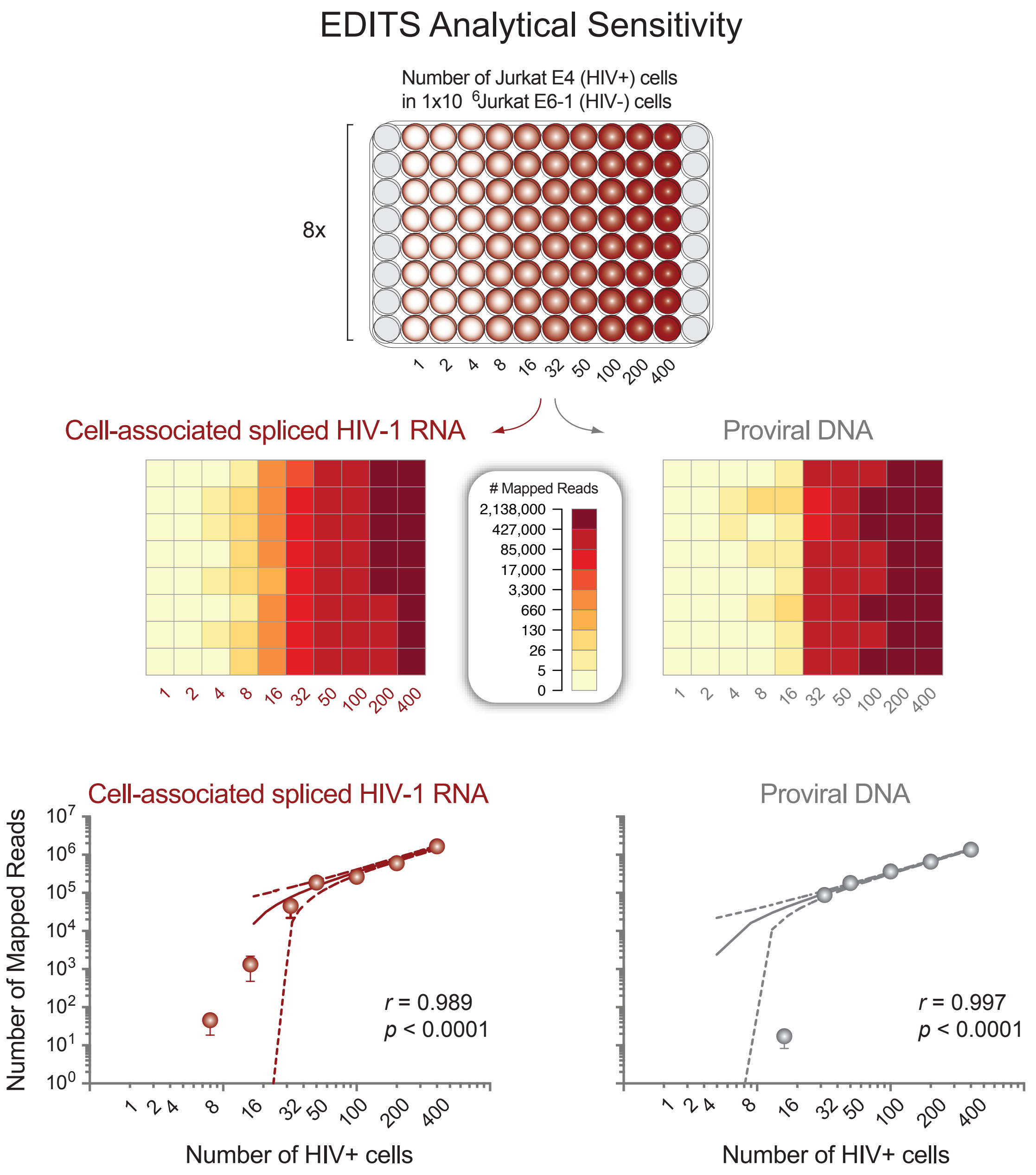

Supplementary Figure 3. Analytical sensitivity of the EDITS assay. Jurkat E4, a cell line latently infected with a single copy of HIV-1 per cell, and Jurkat E6-1, an HIV-negative human T cell lymphoblast, were quantified and serial dilutions were used to prepare ten mixtures containing 0 to 400 Jurkat E4 cells in a background of one million Jurkat E6-1 cells in eight biological replicates. Cell mixtures were activated and cell-associated spliced HIV-1 RNA and proviral DNA quantified using EDITS assay as described in Materials & Methods. Heatmaps indicate the number of HIV-1 vpu/end reads in each of the eight cell mixture replicates. Linear dynamic ranges and regression values (Pearson's coefficient correlation) describing the relationship between mapped vpu/env reads and number of HIV positive cells in the mixtures are indicated. Median mapped reads and interquartile range are depicted.*r*, correlation coeficient; *p*, two-tailed p value.
