# Supplementary material for: Reduced and highly diverse peripheral HIV-1 reservoir in virally suppressed patients infected with non-B HIV-1 strains in Uganda
Source: Retrovirology. 2022 Jan 15;19:1. doi: 10.1186/s12977-022-00587-3 (PMC8760765; doi:10.1186/s12977-022-00587-3)
Supplement: Supplementary file 5 — Additional file 5: Figure S4. Validation of the EDITS assay. Memory CD4+ T cells from five ART-experienced, well-suppressed (plasma HIV-1 RNA load < 20 copies/ml) individuals infected with HIV-1 subtype B strains in Cleveland, OH were used to quantify the peripheral HIV-1 reservoir using the EDITS assay, proviral DNA deep sequencing assay, and a dPCR test designed to quantify proviral DNA as described in “Methods” section. Linear dynamic ranges and regression values (Pearson’s coefficient correlation) describing the relationship between the size of the HIV-1 reservoir quantified by each assay are indicated. r, correlation coeficient; p, two-tailed p value. [file 12977_2022_587_MOESM5_ESM.pdf]

EDITS Validation with dPCR

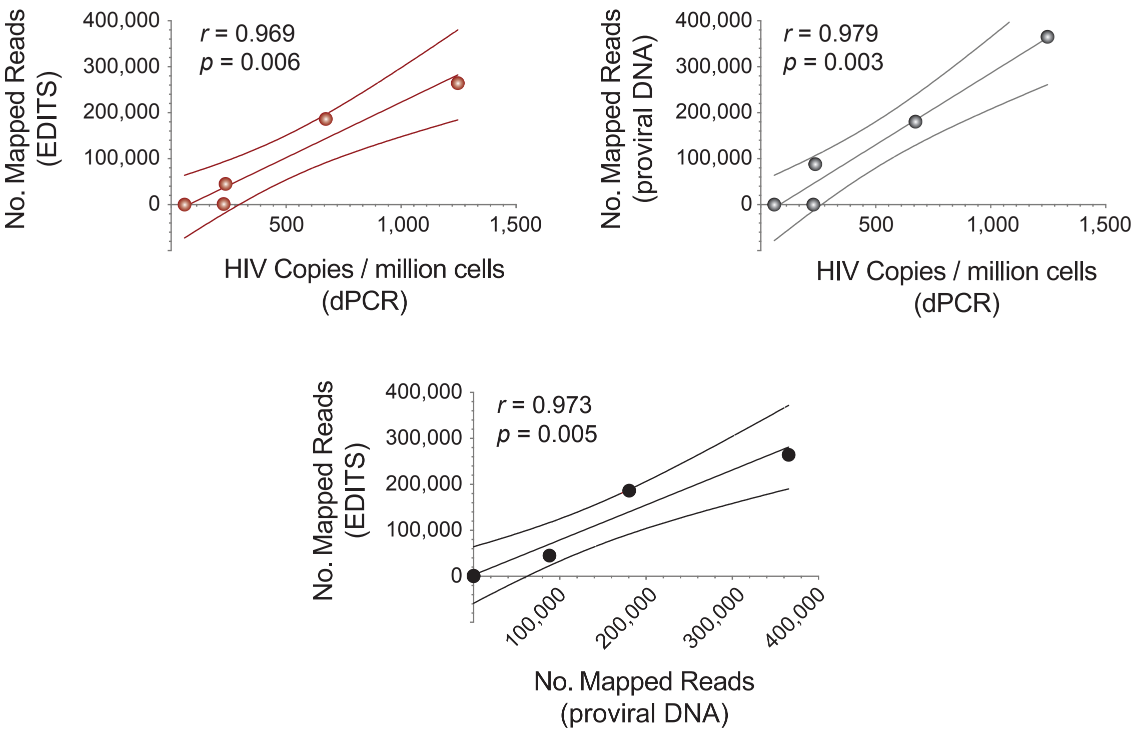

Supplementary Figure 4. Validation of the EDITS assay. Memory CD4+ T cells from five ART-experienced, well-suppressed (plasma HIV-1 RNA load <20 copies/ml) individuals infected with HIV-1 subtype B strains in Cleveland, OH were used to quantify the peripheral HIV-1 reservoir using the EDITS assay, proviral DNA deep sequencing assay, and a dPCR test designed to quantify proviral DNA as described in Materials & Methods. Linear dynamic ranges and regression values (Pearson's coefficient correlation) describing the relationship between the size of the HIV-1 reservoir quantified by each assay are indicated. *r*, correlation coeficient; *p*, two-tailed p value.
