# Supplementary material for: Reduced and highly diverse peripheral HIV-1 reservoir in virally suppressed patients infected with non-B HIV-1 strains in Uganda
Source: Retrovirology. 2022 Jan 15;19:1. doi: 10.1186/s12977-022-00587-3 (PMC8760765; doi:10.1186/s12977-022-00587-3)
Supplement: Supplementary file 6 — Additional file 6: Figure S5. Relevant metrics generated during each one of the deep sequencing runs corresponding to the EDITS assay and/or the proviral DNA test. Number of samples included in each specific MiSeq run. Clusters Passing Filter (Cluster PF) and Total Reads Passing Filters (Total Reads PF), represent the percentage of generated clusters and number of reads, respectively, that passed an internal quality filtering procedure used by Illumina. Cluster Density indicates the amount of clusters that were generated per flow cell surface area during the cluster generation stage. Error rate was calculated based on the PhiX control samples spiked in each deep sequencing run, showing the number of bases with a mismatch relative to the PhiX sequence. Median values and interquartile range are depicted. [file 12977_2022_587_MOESM6_ESM.pdf]

Deep Sequencing Run Metrics

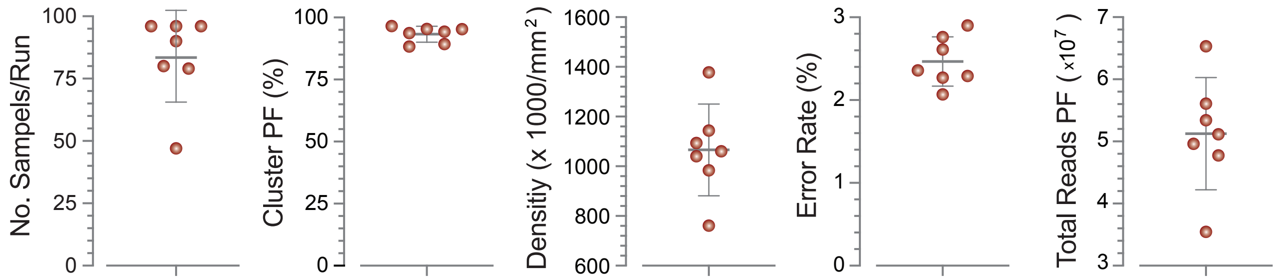

Supplementary Figure 5. Relevant metrics generated during each one of the deep sequencing runs corresponding to the EDITS assay and/or the proviral DNA test. Number of samples included in each specific MiSeq run. Clusters Passing Filter (Cluster PF) and Total Reads Passing Filters (Total Reads PF), represent the percentage of generated clusters and number of reads, respectively, that passed an internal quality filtering procedure used by Illumina. Cluster Density indicates the amount of clusters that were generated per flow cell surface area during the cluster generation stage. Error rate was calculated based on the PhiX control samples spiked in each deep sequencing run, showing the number of bases with a mismatch relative to the PhiX sequence. Median values and interquartile range are depicted.
