# Supplementary material for: Reduced and highly diverse peripheral HIV-1 reservoir in virally suppressed patients infected with non-B HIV-1 strains in Uganda
Source: Retrovirology. 2022 Jan 15;19:1. doi: 10.1186/s12977-022-00587-3 (PMC8760765; doi:10.1186/s12977-022-00587-3)
Supplement: Supplementary file 7 — Additional file 7: Figure S6. Quantification of number of cells used in the EDITS assay. Cellular DNA was extracted from one million memory CD4+ T cells purified from 15 Ugandan and 25 U.S. patients, as well as one million Jurkat E4 (HIV+)/Jurkat E^-1 (HIV-) cells from five points of the standard curve. One microlliter of cellular DNA was use to PCR amplify a 197 bp fragment of the cellular albumin gene. Amplicons were purified and quantified using Qubit 2.0 (Thermo Fisher Scientific). Unpaired t test was used to compare the concentration of albumin amplicon (ng/ul) between both cohort of patients and the cell mixture from the standard curve. Median values and interquartile range are depicted. [file 12977_2022_587_MOESM7_ESM.pdf]

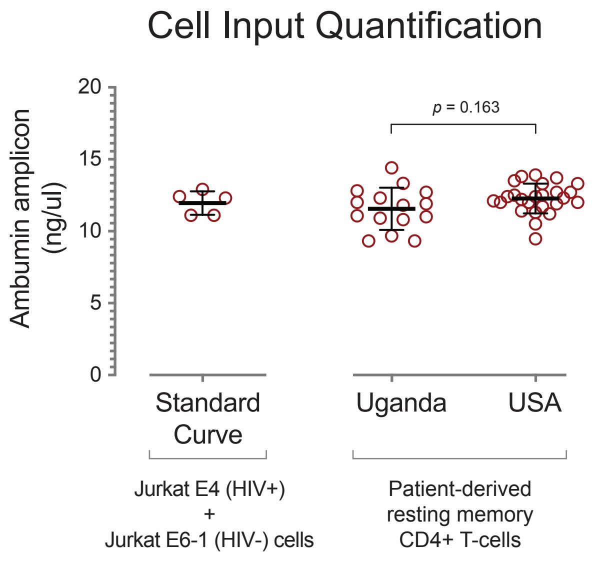

Supplementary Figure 6. Quantification of number of cells used in the EDITS assay. Cellular DNA was extracted from one million memory CD4+ T cells purified from 15 Ugandan and 25 U.S. patients, as well as one million Jurkat E4 (HIV+)/Jurkat E^-1 (HIV-) cells from five points of the standard curve. One microlliter of cellular DNA was use to PCR amplify a 197 bp fragment of the cellular albumin gene. Amplicons were purified and quantified using Qubit 2.0 (Thermo Fisher Scientific). Unpaired t test was used to compare the concentration of albumin amplicon (ng/ul) between both cohort of patients and the cell mixture from the standard curve. Median values and interquartile range are depicted.
