# Supplementary material for: Reduced and highly diverse peripheral HIV-1 reservoir in virally suppressed patients infected with non-B HIV-1 strains in Uganda
Source: Retrovirology. 2022 Jan 15;19:1. doi: 10.1186/s12977-022-00587-3 (PMC8760765; doi:10.1186/s12977-022-00587-3)
Supplement: Supplementary file 8 — Additional file 8: Figure S7. Size of the inducible peripheral HIV-1 reservoir in male and female HIV-infected individuals from Uganda (n = 62) and the U.S. (n =50) estimated by measuring cell-associated spliced HIV-1 RNA (EDITS assay) and proviral DNA. Unpaired t test was used to compare the reservoir size (number of cell equivalents per one million cells) between the different groups of patients. *p < 0.05, **p <0.01, ***p <0.001, ****p <0.0001. Median cell equivalents/million cells and interquartile range are depicted. ♂ male; ♀ female. [file 12977_2022_587_MOESM8_ESM.pdf]

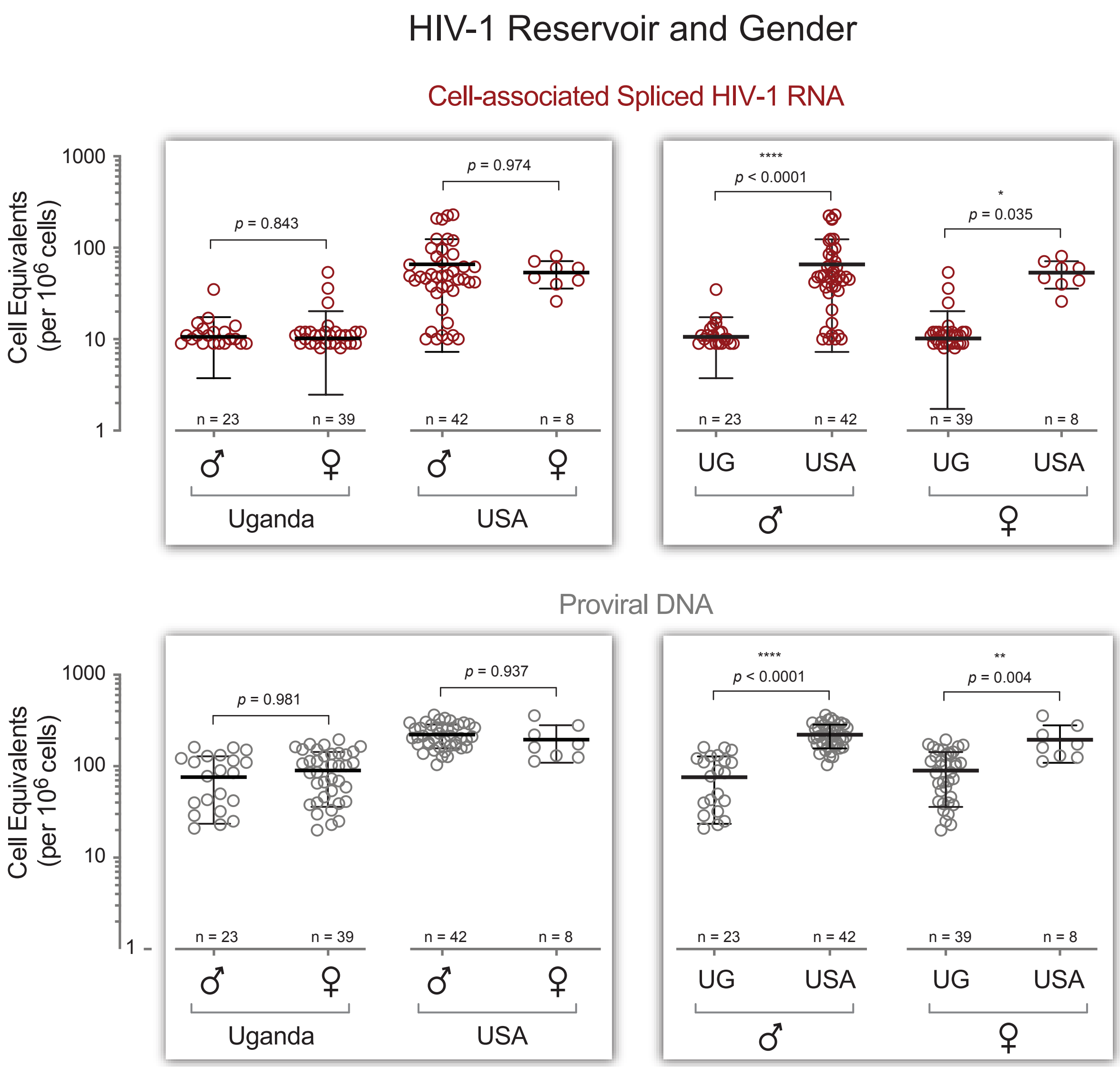

Supplementary Figure 7. Size of the inducible peripheral HIV-1 reservoir in male and female HIV-infected individuals from Uganda (n = 62) and the U.S. (n =50) estimated by measuring cell-associated spliced HIV-1 RNA (EDITS assay) and proviral DNA. Unpaired t test was used to compare the reservoir size (number of cell equivalents per one million cells) between the different groups of patients. * = p <0.05, ** = p <0.01, *** = p <0.001, **** = p <0.0001. Median cell equivalents/million cells and interquartile range are depicted. ♂, male; ♀, female.
